# Viral Control of Mitochondrial Apoptosis

**DOI:** 10.1371/journal.ppat.1000018

**Published:** 2008-05-30

**Authors:** Lorenzo Galluzzi, Catherine Brenner, Eugenia Morselli, Zahia Touat, Guido Kroemer

**Affiliations:** 1 INSERM, U848, Villejuif, France; 2 Institut Gustave Roussy, Villejuif, France; 3 Faculté de Médecine, Université Paris-Sud 11, Villejuif, France; 4 University of Versailles/St Quentin, PRES UniverSud Paris, CNRS UMR8159, Versailles, France; University of British Columbia, Canada

## Abstract

Throughout the process of pathogen–host co-evolution, viruses have developed a battery of distinct strategies to overcome biochemical and immunological defenses of the host. Thus, viruses have acquired the capacity to subvert host cell apoptosis, control inflammatory responses, and evade immune reactions. Since the elimination of infected cells via programmed cell death is one of the most ancestral defense mechanisms against infection, disabling host cell apoptosis might represent an almost obligate step in the viral life cycle. Conversely, viruses may take advantage of stimulating apoptosis, either to kill uninfected cells from the immune system, or to induce the breakdown of infected cells, thereby favoring viral dissemination. Several viral polypeptides are homologs of host-derived apoptosis-regulatory proteins, such as members of the Bcl-2 family. Moreover, viral factors with no homology to host proteins specifically target key components of the apoptotic machinery. Here, we summarize the current knowledge on the viral modulation of mitochondrial apoptosis, by focusing in particular on the mechanisms by which viral proteins control the host cell death apparatus.

## Introduction

The sacrifice, via programmed cell death (PCD), of infected cells represents the most primordial response of multicellular organisms to viruses. This response is common to all metazoan phyla, including plants (which lack an immune system based on mobile cells) (Text S2, [S1]). In mammals, microbial invasion does not only trigger PCD of infected cells but also elicits an immune reaction. This is hierarchically organized in a first-line response provided by innate immune effectors (e.g., infiltrating phagocytes and natural killer cells) [S2], followed by the activation of adaptive immunity, mediated by T and B lymphocytes [S3]. Importantly, other layers of defense exist to prevent viral replication and spread [S2]. For instance, in invertebrates like *Drosophyla melanogaster* (as well as in plants), a prominent antiviral mechanism is provided by RNA interference (RNAi) [S4]. Although the RNAi pathway is preserved in mammals, it has presumably been superseded in its antiviral role by the extremely potent interferon system, as well as by a number of additional mechanisms [S5]. Such a multivariate antiviral response is designed to recognize virions, virus-infected cells, and virus-induced signals of stress (including cell death) to eliminate the pathogen (together with the host cell) and to elicit immunological memory [S6]. Thus, the co-evolution between host and virus has forced the latter to develop strategies for modulating host cell PCD and/or for avoiding immunogenic cell death.

Apoptosis is an active mode of PCD exhibiting a series of morphological and biochemical changes by which it can be distinguished from other cell death subroutines [S7]. At a morphological level, these modifications include a dramatic reduction in cell volume (cell shrinkage), nuclear pyknosis (chromatin condensation), and karyorrhexis (nuclear fragmentation). Eventually, dying cells break down into small, discrete bodies known as apoptotic bodies [S7]. The morphological appearance of apoptosis is accounted for by an ensemble of biochemical events that include, but are not limited to: **(1)** loss of the structural integrity and bioenergetic functions of mitochondria, **(2)** cascade activation of a specific set of catabolic enzymes (e.g., proteases of the caspase family, nucleases), **(3)** exposure of phosphatydylserine on the outer leaflet of plasma membrane and, finally, **(4)** loss of the barrier function of the plasma membrane [S7].

**Figure 1:**
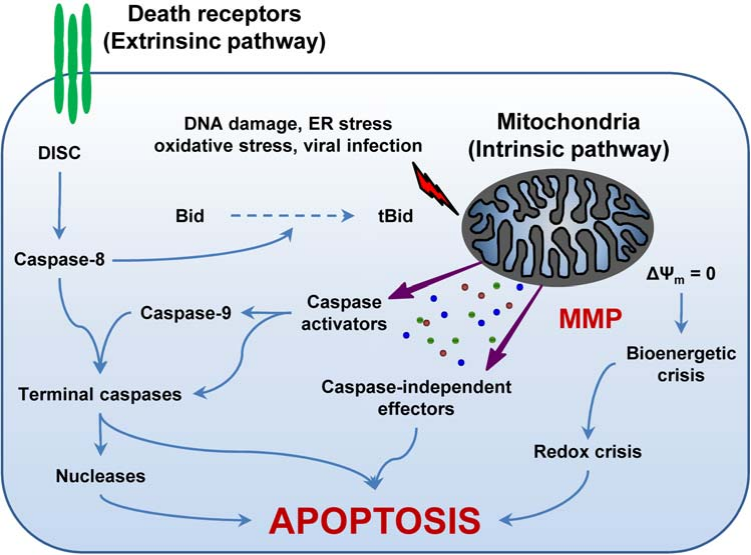
The Extrinsic and the Intrinsic (Mitochondrial) Pathways of Apoptosis. The extrinsic apoptotic pathway involves the activation of death receptors at the cell surface, followed by a caspase cascade that eventually leads to the execution of cell death. In contrast, different proapoptotic stimuli initiate the intrinsic pathway by triggering mitochondrial membrane permeabilization (MMP). Following MMP, intermembrane space proteins are released into the cytosol, the mitochondrial transmembrane potential (Δψ_m_) is dissipated, and the bioenergetic and redox-detoxifying functions of mitochondria are compromised. The resulting bioenergetic and redox crises, associated with the activation of both caspase-dependent and -independent executioner mechanisms, commit the cell to death. The two pathways are interconnected by the BH3-only protein Bid, whose truncated form (tBid) is generated by caspase-8 and can target mitochondria to trigger MMP. For a more detailed description of the intrinsic and extrinsic pathways of apoptosis please refer to the Introduction and to [1]. DISC, death-inducing signaling complex; ER, endoplasmic reticulum.

Apoptosis can be triggered by two fundamentally distinct signaling cascades, namely the extrinsic and intrinsic (or mitochondrial) pathways (for a recent review, see [1]) (Figure 1). The extrinsic pathway is started by the ligand-induced oligomerization of specific cell surface receptors, such as Fas/CD95 and the tumor necrosis factor receptor (TNFR). This induces the intracellular assembly of the death-inducing signaling complex (DISC), a molecular platform for the activation of the caspase cascade that emanates from caspase-8 and results in the activation of effector caspases and nucleases (e.g., caspase-3, -6, and -7, caspase-activated DNase) (Figure 1) [S8,S9]. In contrast, the intrinsic pathway is controlled by mitochondria, which collect and integrate pro- and antiapoptotic signals incoming from other organelles as well as from the extracellular microenvironment. Notably, proapoptotic stimuli as diverse as DNA damage, endoplasmic reticulum (ER) stress, lysosomal stress, reactive-oxygen species (ROS), and calcium (Ca^2+^) overload are able to activate the intrinsic pathway of apoptosis by favoring mitochondrial membrane permeabilization (MMP) (Figure 1) [S10,S11]. In some cells, mitochondrial apoptosis may ensue the activation of death receptors, due to the MMP-promoting activity of the BH3-only protein Bid, which can be proteolytically activated by caspase-8 [S9,S12].

MMP culminates in the loss of mitochondrial transmembrane potential (Δψ_m_), an arrest of mitochondrial bioenergetic and biosynthetic functions, and in the release of mitochondrial intermembrane space (IMS) proteins into the cytosol. These proteins include caspase activators like cytochrome *c* (Cyt *c*) [S13,S14] and Smac/DIABLO [S15], as well as caspase-independent death effectors such as apoptosis-inducing factor (AIF) [S16,S17] and endonuclease G (EndoG) [S18]. Thus, MMP activates caspase-dependent and/or -independent mechanisms to execute cell death [1].

Impaired MMP is associated with multiple pathological conditions, including autoimmune diseases and cancer [S19]. Conversely, unscheduled MMP contributes to the development of diseases characterized by an excess of cell death, such as ischemia/reperfusion injuries, trauma, toxic/metabolic syndromes as well as chronic neurodegenerative conditions like amyotrophic lateral sclerosis or Alzheimer, Parkinson, and Huntington diseases [S20]. MMP is regulated by a complex network of signaling pathways that involves both endogenous (e.g., pro- and antiapoptotic Bcl-2 family proteins [S21,S22], p53 [2;S23,S24], kinases [S25–S28], phosphatases [3;S29,S30], lipid second messengers [4;S31–S33], ROS [5;S34–S37], Ca^2+^ overload [6;S38–S40]) as well as exogenous factors (e.g., viral proteins [7,8;S41,S42], toxins [9;S43–S45], pro-oxidants [S46–S49]) (for reviews, see [1;S10]). As MMP delimits the point of no return in the intrinsic apoptotic cascade, any viral factor that influences MMP must have a major impact on cell fate, either by inducing or blocking cell death [7;S42].

During the last decade, major efforts have been dedicated to the elucidation of the mechanisms underlying MMP in health and disease. According to current beliefs, MMP is executed via either of two distinct, yet partially overlapping and non-mutually exclusive, mechanisms. These two routes to MMP are initiated at different mitochondrial subcompartments (notably at the mitochondrial outer or inner membrane, i.e., OM and IM) and each relies on a specific set of factors. Nonetheless, they both lead to a functional and structural collapse of mitochondria that commits the cell to death [1]. Notably, MMP is associated with dramatic changes in the mitochondrial network as well as in mitochondrial ultrastructure, concerning both matrix volume and cristae organization [S50–S52]. How these structural modifications of mitochondria might impact on viral infection, however, remains to be elucidated.

## MMP Regulation by Bcl-2 Family Proteins

The abundant presence of the voltage-dependent anion channel (VDAC) renders OM freely permeable to solutes and small metabolites up to approximately 5 kDa. This cutoff ensures that soluble proteins are retained in the IMS under normal circumstances. The apoptosis-associated drastic increase in OM permeability may originate at the OM itself by means of multiple mechanisms, including **(1)** the assembly of large homo- or hetero-multimeric channels, allowing for the release of IMS proteins, by proapoptotic pore-forming proteins of the Bcl-2 family (e.g., Bax, Bak) [10,11;S53,S54]; **(2)** the destabilization of the lipid bilayer mediated by proapoptotic Bcl-2 family members (e.g., Bax, truncated Bid, i.e., tBid), which results in the priming of mitochondria for the release of IMS proteins [S55–S58]; and **(3)** the induction of the so-called mitochondrial permeability transition (MPT) at the IM, following the interaction between Bax (or tBid) and components of the permeability transition pore complex (PTPC) at the OM [12;S59–S63]. In this latter case, MMP begins and ends at the OM, yet is mediated by an event taking place mainly at the IM, i.e., MPT (see the section “MMP Regulation by the PTPC” for further details). Independently from the specific mechanisms that activate MMP, the Bcl-2 family of proteins exerts a major regulation of this process [S64].

The Bcl-2 family is composed of antiapoptotic multidomain members (e.g., Bcl-2, Bcl-X_L_, Mcl-1), which contain four Bcl-2 homology (BH) domains (BH1-4) [S65], proapoptotic multidomain proteins (e.g., Bax, Bak) [S66], which contain three BH domains (BH1-3), and pro-apoptotic BH3-only proteins (e.g., Bid, Bad) [13]. Due to an additional C-terminal domain, some members of all the subgroups share the ability to insert into the OM and other intracellular membranes (e.g., ER) [1].The specific set of BH domains contained in each Bcl-2 family member determines its profile of activity [S67–S69]. In this context, early structure-function studies identified BH1, BH2, and BH4 as the major antiapoptotic determinants of Bcl-2 [S67–S70]. Conversely, the presence of the BH3 domain was found to suffice for apoptosis induction by Bax (as well as for heterodimerization with Bcl-2/Bcl-X_L_) [S71–S72]. Later, numerous reports showed that the conserved transmembrane (TM) domain and less conserved, unstructured loops between BH domains also contribute to define the functional profile of Bcl-2 proteins, either by acting as targeting signals for subcellular compartments (e.g., mitochondria, ER) or by modulating the overall tertiary structure [S73–S77].

**Figure 2:**
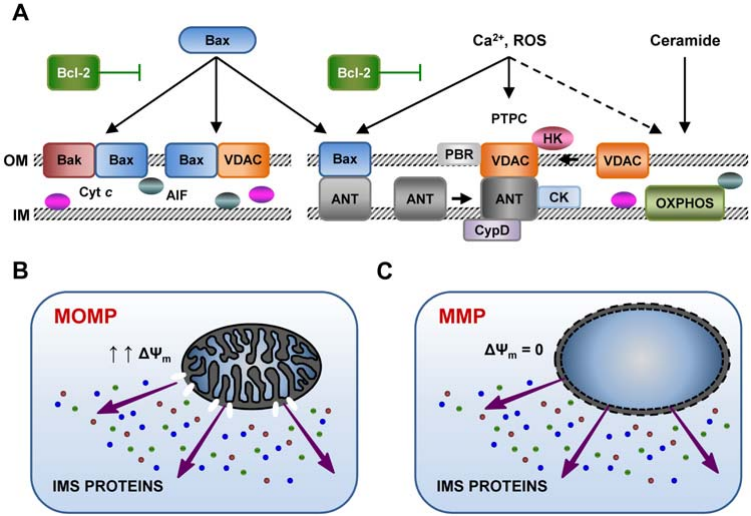
Different Models of Mitochondrial Membrane Permeabilization (MMP). Large pores formed by the oligomerization of proapoptotic Bcl-2 proteins (e.g., Bax, Bak) and/or the voltage-dependent anion channel (VDAC) may promote selectively mitochondrial outer membrane permeabilization (MOMP). In this case, specific intermembrane space (IMS) proteins are liberated in the cytosol, but the mitochondrial transmembrane potential (Δψ_m_) is (at least initially) retained (A,B). On the contrary, some proapoptotic stimuli, such as calcium (Ca^2+^) overload, reactive oxygen species (ROS), and the lipid second messenger ceramide, favor MMP by inducing the permeabilization of the inner mitochondrial membrane (IM) via the activation of the permeability transition pore complex (PTPC). When the PTPC opens, Δψ_m_ is immediately lost and an unregulated entry of solutes and water into the mitochondrial matrix occurs. This results in the osmotic swelling of mitochondria, followed by rupture of both mitochondrial membranes and the unspecific release into the cytosol of IMS proteins (A,C) (please refer to the sections “MMP Regulation by Bcl-2 Family Proteins” and “MMP Regulation by the PTPC” for additional details). Notably, antiapoptotic proteins from the Bcl-2 family play a role in both models. AIF, apoptosis-inducing factor; ANT, adenine nucleotide translocase; CK, creatine kinase; CypD, cyclophilin D; Cyt *c*, cytochrome *c*; HK, hexokinase; OXPHOS, oxidative phosphorylation complexes; PBR, peripheral-type benzodiazepine receptor.

Bcl-2/Bcl-X_L_ stabilizes mitochondrial membranes via multiple mechanisms, including **(1)** the sequestration into inactive complexes of its proapoptotic counterparts, Bax, Bak, and BH3-only proteins (e.g., Bid) (for review: [S65,S78]), **(2)** inhibitory interactions with PTPC constituents, in particular with VDAC and the adenine nucleotide translocase (ANT) [12],[14], **(3)** an enhancement of Cyt *c* oxidase activity and mitochondrial respiration [S79], and/or **(4)** indirect effects on intracellular Ca^2+^ stores of the ER [S80,S81]. While Bax/Bak execute MMP by one (or more) of the aforementioned mechanisms, BH3-only proteins exhibit indirect proapoptotic effects [1;S66,S82]. Thus, “activator” BH3-only proteins (e.g., Bid) would directly interact and activate Bax/Bak, whereas the “derepressors” (e.g., Bad) would rather disrupt Bcl-2/Bcl-X_L_ inhibitory complexes, thus allowing for the release of Bax/Bak [15,S82]. In healthy cells, inactive Bak is constitutively associated with mitochondria, while Bax is found as a cytosolic monomer [S66]. Upon apoptosis induction, both undergo a conformational modification to become activated, and Bax translocates to OM in the form of an active dimer [S83,S84]. When Bax or Bak induce MMP, the release of specific IMS proteins occurs via large pores in the OM and may precede Δψ_m_ loss. In some instances of Bax-mediated apoptosis, indeed, mitochondrial membrane permeabilization (MOMP) occurs without discernable Δψ_m_ alterations [1] (Figure 2).

Despite their structural similarity, each BH3-only protein presents a specific mechanism of activation (either at a transcriptional level or mediated by post-translational modifications), and acts as a sensor of a particular type of cell stress [15,S82]. For instance, the BH3-only proteins Puma and Noxa are activated by DNA damage via p53-dependent transactivation [16;S85], Bim and Bmf are released from cytoskeletal structures upon c-Jun N-terminal kinase (JNK)-mediated phosphorylation [S86–S88], and Bid is proteolytically processed by caspase-8 following the activation of the extrinsic pathway of apoptosis [S9,S12]. Following caspase-8-mediated cleavage, a glycine residue of tBid is exposed, allowing for post-translational (rather than usual cotranslational) N-myristoylation. This modification has been shown to act as an activating switch and to enhance tBid-induced Cyt *c* release and cell death [S89]. Interestingly, tBid mitochondrial targeting [S90] and proapoptotic activity [S91] have been associated with cardiolipin, a mitochondrial lipid particularly abundant in the IM. tBid-cardiolipin interaction requires three α-helical domains (α4-α6) of tBid and occurs prominently at the contact sites between the OM and the IM [S92]. Cardiolipin might also be implicated in the dissociation of Bid fragments (tBid and nBid), which would rather occur during the targeting of tBid to mitochondria than immediately after caspase-8-mediated cleavage [S93]. Although Bcl-2 family proteins exert their apoptosis-modulatory functions mainly at mitochondria, extra-mitochondrial activities contribute to their effects. For instance, Bcl-2/Bcl-X_L_ localize at the ER and decrease luminal Ca^2+^ concentration, thus protecting against Ca^2+^-dependent death stimuli [S80,S94,S95]. Conversely, Bax/Bak favor the transfer of Ca^2+^ from the ER to mitochondria and cell death [S96,S97]. Moreover, *bax^−/−^/bak^−/−^* MEFs show an impaired mobilization of ER Ca^2+^ following numerous proapoptotic stimuli, which can be partially restored by overexpressing the sarco/endoplasmic reticulum Ca^2+^ ATP-ase (SERCA) [17]. Taken altogether, these observations point to the Bcl-2 system as a prominent pharmacological target for the modulation of mitochondrial apoptosis (for a review, see [18]).

## MMP Regulation by the PTPC

MMP may originate at the IM due to the activation of the PTPC, a large multiprotein structure assembled at the contact sites between OM and IM. This applies in particular to cell death models characterized by enhanced Ca^2+^ fluxes and disproportionate ROS generation [S98]. PTPC activation provokes a sudden increase in the IM permeability to solutes of low molecular weight (i.e., MPT), which leads to the unregulated entry of water and osmotic swelling of the mitochondrial matrix. In turn, this may result in the physical rupture of the OM, because the surface area of the IM (with its folded cristae) largely exceeds that of the OM [S63,S98,S99]. In the context of MPT-derived MMP, Δψ_m_ dissipates before OM is permeabilized and IMS are released (Figure 2) [S63]. Although its exact molecular composition remains elusive, numerous independent studies suggest that the PTPC might result from the association of multiple proteins, including ANT (in the IM) and VDAC (in the OM), in the context of a dynamic interaction with mitochondrial matrix proteins (e.g., cyclophilin D [CypD]), IMS proteins (e.g., creatine kinase [CK]), OM proteins (e.g., peripheral-type benzodiazepine receptor [PBR]), as well as with cytosolic factors (e.g., hexokinase isoforms) (for recent reviews, see [S63,S100,S101]). Nevertheless, genetic studies performed in the murine system suggest that all the aforementioned components of the PTPC, most of which exist in multiple isoforms, are either dispensable for cell death or preferentially participate in necrotic pathways (rather than in apoptosis) [19–21;S102].

In addition, controversial views remain about the mechanisms by which the PTPC promotes MPT and therefore MMP. Some authors have proposed that in physiological conditions VDAC would be found within the PTPC in a state of low conductance, rapidly switching between the open and closed conformations [S103]. In this configuration, the PTPC would ensure the normal exchange of metabolites between the mitochondrial matrix and the cytosol. Following proapoptotic stimuli, a state of high conductance for VDAC would be favored, resulting in long-lasting openings of the PTPC and MPT [12;S59]. Alternatively, it has been suggested that the high conductance state of VDAC would serve to its physiological functions, whereas cell death would result from a closed conformation, favoring a transient hyperpolarization of the mitochondrial matrix, followed by osmotic imbalance, swelling, and eventually MMP [S104–S105].

Several reports indicate PTPC components as targets for the apoptosis-modulatory activity of both pro- and antiapoptotic Bcl-2 family members. In this context, it has been demonstrated that Bax and Bak accelerate the opening of VDAC in reconstituted proteoliposomes, and that VDAC-deficient mitochondria do not exhibit Bax/Bak-induced Cyt *c* release and Δψ_m_ dissipation occurring in VDAC-proficient control mitochondria [12;S106]. In the same model, recombinant Bcl-2/Bcl-X_L_ as well as synthetic peptides corresponding to their BH4 domains were shown to prevent VDAC opening, Cyt *c* release, and Δψ_m_ dissipation [S107,S108]. In addition, the BH3-only proteins Bid and Bim have been reported to interact directly with VDAC, the latter interaction being remarkably enhanced during apoptosis [S62,S109].

Bax and Bcl-2/Bcl-X_L_ also modulate PTPC activity by binding to ANT. As demonstrated by the yeast two-hybrid system, co-immunoprecipitation assays, and in artificial lipid bilayers, ANT and Bax directly interact and cooperate to form a channel with distinct electrophysiological properties as compared to the channels formed by Bax or ANT alone [22;S110]. In artificial membranes, the presence of Bcl-2 inhibited cooperative channel formation by Bax and ANT as well as the atractyloside-induced assembly of channels by ANT alone, thus pointing to a direct interaction between ANT and Bcl-2 [S46,S110]. Furthermore, Bcl-2 promotes (and Bax inhibits) ADP/ATP exchange in ANT-containing proteoliposomes, isolated mitochondria, and mitoplasts [S111]. Interestingly, in this system the Bax-mediated inhibition of ANT translocase activity could be separated from the formation of cooperative channels by Bax and ANT [S111]. As determined by co-immunoprecipitation and proteomics analysis, the interactome of ANT undergoes major rearrangements in the course of the chemotherapy-induced apoptosis. Thus, soon after the treatment with etoposide of a human tumor cell line (HT29 cells), the amount of Bax contained within the ANT interactome significantly augmented, whereas the quantity of Bcl-2 was decreased [S112].

As previously mentioned in the section “MMP Regulation by Bcl-2 Family Proteins”, Bcl-2 family members are known to modulate luminal Ca^2+^ concentration and Ca^2+^ release at the ER [17;S94,S95]. In doing so, they exert an additional indirect control on the PTPC, since cytosolic Ca^2+^ liberated from ER stores (for instance upon the induction of the unfolded protein response) can accumulate in mitochondria and promote PTPC opening, MPT-dependent MMP, and cell death [S40]. Thus, it appears that an intricate crosstalk for the modulation of MMP exists between mitochondria and the ER, in which proteins from the Bcl-2 family participate at the level of both organelles [S80,S81].

## Viral Modulation of Mitochondrial Apoptosis

**Figure 3:**
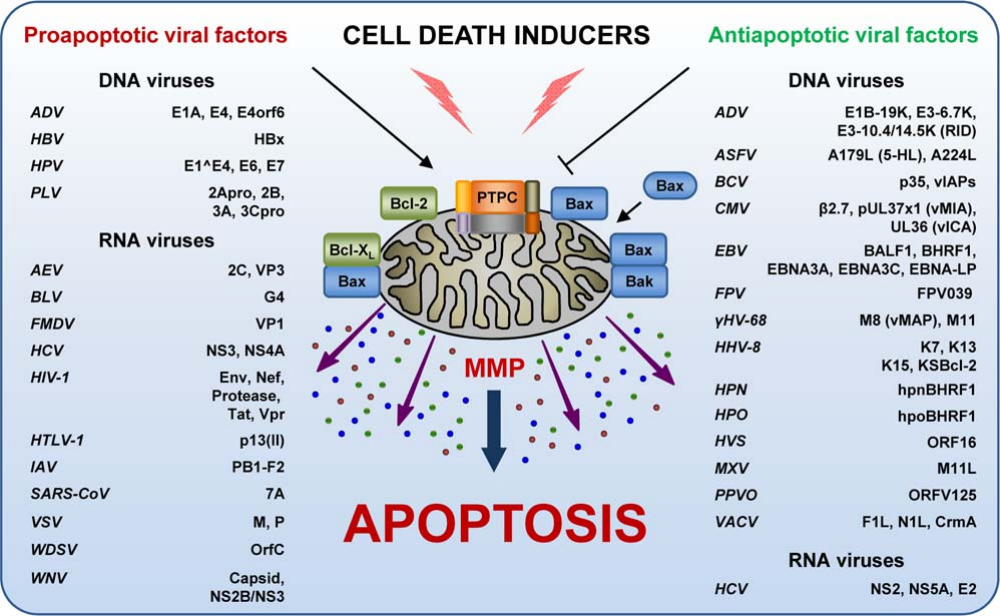
Control of Mitochondrial Membrane Permeabilization (MMP) by Viral Proteins. A number of viral polypeptides modulate apoptosis, either by favoring or inhibiting MMP. This control can be exerted directly at mitochondria, or on upstream/downstream steps of the apoptotic cascade (please refer to the section “Viral Modulation of Mitochondrial Apoptosis” for further details). ADV, adenovirus; AEV, avian encephalomyelitis virus; ASFV, African swine fever virus; BLV, bovine leukemia virus; BCV, baculovirus; CMV, cytomegalovirus; EBNA, Epstein–Barr nuclear antigen; EBV, Epstein–Barr virus; Env, envelope glycoprotein complex; FMDV, foot-and-mouth disease virus; FPV, fowlpox virus; γHV-68, γ-herpesvirus 68; HBV, hepatitis B virus; HBx, HBV X protein; HCV, hepatitis C virus; HHV-8, human herpesvirus 8; HIV-1, human immunodeficiency virus 1; HPN, herpesvirus pan; HPO, herpesvirus papio; HPV, human papillomavirus; HTLV-1, human T lymphotropic virus 1; HVS, herpesvirus saimiri; IAV, influenza A virus; KSBcl-2, Kaposi sarcoma Bcl-2; M, matrix protein; MXV, myxoma virus; NS, non structural protein; ORF, open reading frame; P, phosphoprotein P; PPVO, parapoxvirus ORF virus; PTPC, permeability transition pore complex; PLV, poliovirus; RID, receptor internalization and degradation complex; SARS-CoV, severe acute respiratory syndrome coronavirus; VACV, vaccinia virus; vIAPs, viral inhibitor of apoptosis proteins; vICA, viral inhibitor of caspase-8 activation; vMAP, viral mitochondrial antiapoptotic protein; Vpr, viral protein R; VSV, vesicular stomatitis virus; WDSV, Walleye dermal sarcoma virus; WNV, West Nile virus.

During the last decade, numerous viral proteins have been reported to modulate (either positively or negatively, either in a direct or indirect fashion) the apoptotic response of host cells to infection (Figure 3, Tables 1 and 2) [7;S42]. With regard to this, viral factors can be classified into one of the four following subgroups: proapoptotic proteins **(1)** that insert into mitochondrial membranes and hence trigger MMP through the action of amphipathic α-helical domains or **(2)** that promote MMP indirectly, through the activition of host-encoded factors (Table 1), and antiapoptotic modulators **(3)** that exhibit sequence and/or structural similarity to multidomain BH1-4 members of the Bcl-2 family (so-called viral Bcl-2 proteins [vBcl-2s]) or **(4)** that inhibit apoptosis via other mechanisms (Table 2). Notably, some viral proteins exhibit mixed apoptosis-modulatory functions, and hence cannot be unambiguously classified into one of the aforementioned groups.

**Table 1:** Examples of Viruses and Viral Proteins Activating the Mitochondrial Pathway of Apoptosis.

Virus	Effector	Cellular Target	Effects	Reference
***Direct inducers of MMP***				
**AEV**	2C	CEB and Cos-7 cells	Induction of the caspase cascade leading to apoptosis	[S154]
	VP3	Different cell lines	Induction of the caspase cascade leading to apoptosis	[S153]
**BLV**	G4	HeLa and yeast cells	Alterations of mitochondrial morphology; interacts with FPPS	[S140]
**HBV**	HBx	Hepatocytes VDAC	Δψ_m_ loss and MMP-dependent apoptosis	[S122–S124]
**HCV**	NS4A	Hepatocytes	Accumulation in mitochondria, Δψ_m_ loss, Cyt *c* release, casp-3 activation	[S155]
**HIV-1**	Vpr	CD4^+^ lymphocytes ANT/VDAC	Δψ_m_ loss, IMS proteins release, caspase cascade activation	[24;S41,S113,S115]
**HTLV-1**	p13(II)	Lymphocytes	Rapid flux of K+ and Ca^2+^ across IM, swelling, Δψ_m_ dissipation and fragmentation	[S135,S138]
**HPV**	E1̂E4	Keratinocytes	Displaces mitochondria from microtubules	[S152]
**IAV**	PB1-F2	Lung cells ANT3/VDAC1	Interaction with ANT3 and VDAC1	[S149]
**PLV**	Unknown	Neurons Bone marrow Lymphoid cells	JNK activation, Cyt *c* release, Δψ_m_ loss, Bax activation	[S176]
	2B 3A	Kidney cells	Perinuclear aggregation and ultrastructural alterations of mitochondria, Δψ_m_ loss	[S130]
**WDSV**	OrfC	Epithelial cells	Perinuclear clustering of mitochondria, Δψ_m_ loss, partial Cyt *c* release, signs of apoptosis	[S131,S132]
***Indirect MMP Facilitators***				
**ADV**	E1A E4orf6	Epithelial cells	Inhibition of PP2A, PARP hyperactivation, AIF translocation; sensitization to TNFα and Fas; BH3-only protein expression	[41;S183,S187]
**EBV**	BALF1	Lymphoid cells	Antagonism of BHRF1(?)	[67]
**FMDV**	Unknown	Thyroid cells Kidney cells Dendritic cells	Reduction in the endogenous levels of Bcl-2, activation of caspases, release of Cyt *c* from mitochondria	[S206]
**HCV**	NS3	Hepatocytes	Casp-8-mediated apoptosis	[S171]
**HIV-1**	Env	CD4^+^ lymphocytes	Bax upregulation, Cyt *c* release, caspase activation; involvement of Cdk1, mTOR, p53 and NF-kB	[32,33;S160]
	Nef	Lymphocytes	Lysosomal membrane permeabilization, cathepsin D release, Bax activation, MMP-dependent cell death	[S158,S159]
	Protease	Lymphocytes Bcl-2/Casp-8	Bcl-2 depletion, NF-kB activation; Casp-8 activation, Bid cleavage, Cyt *c* release, Casp-9 and -3 activation, DFF and PARP cleavage	[36;S169]
	Tat	Neurons Tubulin	Accumulation at mitochondria, Δψ_m_ loss, ROS overproduction, caspase activation; degradation of MAP2; Bim activation; regulation of p53, Bax and Bcl-2	[34,35;S86,S161–S163,S165–S166]
**HPV**	E6	Epithelial cells	Sensitize cells to different apoptotic stimuli, via mechanisms that may depend or not on p53	[S191–S193]
	E7	Epithelial cells	Sensitization of cells to apoptosis induced by growth factor withdrawal, chemotherapeutic agents and UV rays	[S194–S196]
**PLV**	2Apro 3Cpro	HeLa cells	Caspase-dependent apoptosis	[S172,S173]
**SARS-CoV**	7a	Various cell lines	Inhibition of Bcl-X_L_	[46]
**VSV**	M	Vero cells	Reduction of Bcl-X_L_ levels; caspase activation	[44;S199]
	P	Hamster cells	Unknown mechanism	[S203]
**WNV**	Capsid NS2B/NS3	Neurons	UPR, Bid and Bax translocation to mitochondria, Δψ_m_ dissipation, Cyt *c* release, caspase activation	[45;S207–S210]

Abbreviations: ADV, adenovirus; AEV, avian encephalomyelitis virus; AIF, apoptosis-inducing factor; ANT, adenine nucleotide translocase; BH3, Bcl-2 homology domain 3; BLV, bovine leukemia virus; Casp, caspase; Cdk1, cyclin-dependent kinase 1; CEB, chick embryo brain; Cyt *c*, cytochrome *c*; Δψ_m_, mitochondrial transmembrane potential; DFF, DNA fragmentation factor; EBV, Epstein-Barr virus; Env, envelope glycoprotein complex; FMDV, foot-and-mouth disease virus; FPPS, farnesyl pyrophosphate synthetase; HBV, hepatitis B virus; HBx, HBV X protein, HCV, hepatitis C virus; HIV-1, human immunodeficiency virus 1; HPV, human papillomavirus; HTLV-1, human T lymphotropic virus 1; IAV, influenza A virus; IMS, mitochondrial intermembrane space; JNK, c-Jun N-terminal kinase; M, matrix protein; MAP2, microtubule-associated protein 2; MMP, mitochondrial membrane permeabilization; mTOR, mammalian target of rapamycin; NF-kB, nuclear factor-kappa B; NS; non structural protein; ORF, open reading frame; P, phosphoprotein P; PARP, poly(ADP-ribose) polymerase; PLV, poliovirus; PP2A, protein phosphatase 2A; ROS, reactive oxygen species; SARS-CoV; severe acute respiratory syndrome coronavirus; TNFα, tumor necrosis factor α; UPR, unfolded protein response; UV, ultraviolet; VDAC, voltage-dependent anion channel; Vpr, viral protein R; VSV, vesicular stomatitis virus; WDSV, Walleye dermal sarcoma virus; WNV, West Nile virus.

**Table 2:** Examples of Viruses and Viral Proteins Inhibiting Apoptosis.

Virus	Effector/Target	Cellular Target	Effect	Reference
***Viral Bcl-2 Homologs (vBcl-2s)***				
**ADV**	E1B-19K	Epithelial cells	Sequester multiple proapoptotic Bcl-2-like proteins and p53; inhibits apoptosis triggered by numerous stimuli	[38,48;S177,S217–S219]
**ASFV**	A179L (5-HL)	Lymphoid cells	Inhibits apoptosis induced by growth factor deprivation and chemotherapeutics	[S272,S274]
**CMV**	pUL37x1 (vMIA)	Epithelial cells	Bax inhibition, modulates ER Ca^2+^ release and ATP levels; mitochondrial fragmentation; ANT interaction	[52,54;S228,S231]
**EBV**	BALF1	Lymphoid cells	Interacts with Bak/Bax; protects from serum deprivation	[65],[67]
	BHRF1	Lymphoid cells	Blocks apoptosis by death receptors, *c-myc*, granzyme B, DNA damage, infection, radio- and chemotherapy	[70;S248–S253]
**FPV**	FPV039	Fibroblasts	Bak neutralization	[63]
**γHV-68**	M11	Human, murine and yeast cells	Inhibits Fas- and TNFα-induced apoptosis in mammalian cells; prevents Bax toxicity in yeast	[S260,S265,S267]
**HHV-8**	KsBcl-2	FL5.12 cells	vBcl-2 not interacting with proapoptotic proteins from the Bcl-2 family (e.g., Bax, Bak)	[71]
	K7	Lymphoid cells	Inhibits Casp-3 by bridging it with Bcl-2, modulates intracellular Ca^2+^ and proteasome activity	[76,S270,S271]
**HPN**	hpnBHRF1	Lymphoid cells	Protection against apoptosis induced by serum withdrawal, etoposide and UV irradiation	[S258]
**HPO**	hpoBHRF1	Keratinocytes	Protection against cisplatin-induced apoptosis	[S257]
**HVS**	ORF16	T cells	Heterodimerization with Bax and Bak	[72]
**MXV**	M11L	Skin cells	Structural vBcl-2; blocks both Bak/Bax-dependent MOMP and MPT by interacting with PBR	[58],[59]
**PPVO**	ORFV125	Skin cells	Inhibits Bak/Bax activation; blocks UV-induced apoptosis	[64]
**VACV**	F1L	Skin cells	Interacts with Bak and Bim; inhibition of Bax activation at upstream levels	[S234,S235]
	N1L	Skin cells	Structural vBcl-2; inhibits multiple proapoptotic Bcl-2-like proteins (e.g., Bid, Bax, Bak, Bad)	[62]
***Other Antiapoptotic Viral Strategies***				
**ADV**	E3-10.4K/14.5K (RID)	Epithelial cells	Inhibits the extrinsic pathway of apoptosis by favoring the internalization and degradation of death receptors	[87;S219,S299–S303]
**ASFV**	A224L (vIAP)	Macrophages	Casp-3 inhibition; NF-kB activation	[97;S322]
**BCV**	p35	Most cell types	Widely acting inhibitor of metazoan caspases; inhibits also oxidant-induced apoptosis upstream of caspases	[89;S312,S313,S319]
	vIAP	Insect cells	Direct inhibitor of effector caspases	[S320]
**CMV**	UL36 (vICA)	Epithelial cells	Inhibition of Casp-8	[78;S275]
**EBV**	EBNA3A EBNA3C	Lymphoid cells	Downregulation of Bim	[S277]
	EBNA-LP	Lymphoid cells	Interaction with Bcl-2 through HAX-1	[80]
**γHV-68**	vMAP	Different cell lines	Recruitment of Bcl-2 at mitochondria, inhibition of Cyt *c* release through interaction with VDAC1	[99]
**HCV**	E2	Hepatocytes	Inhibition of TRAIL-induced apoptosis	[82]
	NS2	Hepatocytes	Inhibition of MMP and apoptosis induced by CIDE-B	[81]
	NS5A	Hepatocytes	Activation of NF-kB, interaction with FKBP38	[S284,S285]
**HHV-8**	K13 (vFLIP)	Lymphoid cells	NF-kB activation	[S296]
	K15	Lymphoid cells	Antiapoptotic function by interaction with HAX-1	[S286]
**VACV**	CrmA	Several cell lines	Serpin, direct inhibitor of caspases	[55]

Abbreviations: ADV, adenovirus; ANT, adenine nucleotide translocase; ASFV, African swine fever virus; BCV, baculovirus; Casp, caspase; CIDE-B, cell-death-inducing DFF-45-like effector B; CMV, cytomegalovirus; Δψ_m_, mitochondrial transmembrane potential; EBNA, Epstein-Barr nuclear antigen; EBV, Epstein-Barr virus; ER, endoplasmic reticulum; FKBP38, 38-kDa FK506-binding protein FPV, fowl poxvirus; γHV-68, γ-herpesvirus 68; HAX-1, HS1-associated protein X-1; HHV-8, human herpesvirus 8; HVS, herpesvirus saimiri; KsBcl-2, Kaposi sarcoma Bcl-2; MMP, mitochondrial membrane permeabilization; MOMP, mitochondrial outer membrane permeabilization; MPT, mitochondrial permeability transition; MXV, Myxoma virus NF-kB, nuclear factor-kappa B; HPN, herpesvirus pan; HPO, herpesvirus papio; ORF, open reading frame; PBR, peripheral-type benzodiazepine receptor; PPVO, parapoxvirus ORF virus; RID, receptor internalization and degradation complex; TNFα, tumor necrosis factor α; UV, ultraviolet; VACV, vaccinia virus; vBcl-2, viral Bcl-2; VDAC, voltage-dependent anion channel; vFLIP, viral Fas-associated death domain-like interleukin 1β converting enzyme (FLICE) inhibitory protein; vIAP, viral inhibitor of apoptosis protein; vICA, viral inhibitor of caspase-8 activation; vMAP, viral mitochondrial antiapoptotic protein; vMIA, viral mitochondrial-localized inhibitor of apoptosis.

## Viral Proapoptotic Proteins

### Direct Inducers of MMP

Several proteins encoded by the human immunodeficiency virus 1 (HIV-1) exert a proapoptotic activity, thereby contributing to the HIV-induced depletion of CD4^+^ lymphocytes (for a review, see [23]). Among these, the viral protein R (**V**pr) has direct mitochondrial effects in numerous cell types independently from its mode of delivery (viral infection, transfection of a *vpr* gene, or exogenous administration of recombinant Vpr protein) [S113,S114]. The C-terminal moiety (aa 52–96) of Vpr directly interacts with ANT and VDAC, thereby triggering MMP associated with Δψ_m_ loss, IMS proteins release, and caspase cascade activation [24;S41,S115]. When added in vitro to purified mouse liver mitochondria, a synthetic Vpr-derived peptide (Vpr_52–96_) induced large amplitude swelling (an indicator of IM permeabilization and PTPC pore opening) in less than 15 minutes. This effect could be prevented by Bcl-2 as well as by pharmacological agents targeting ANT (such as bongrekic acid [BA]) or VDAC (such as 4,4′-diisothiocyanatostilbene-2,2′-disulfonic acid [DIDS]) [24]. In lymphoid cells, Vpr-mediated MMP and apoptosis is facilitated by Bax, yet inhibited by overexpression of Bcl-2 or addition of the PTPC inhibitor cyclosporine A (CsA) [S41,S115]. Recently, it has been proposed that two distinct domains of Vpr (namely aa 27–51 and aa 71–82) would bind to a region encompassing the first ANT IMS loop and part of its second and third TM helices [S114]. This model may explain why Vpr is able to convert ANT into a non-specific pore, as this has been observed experimentally when adding Vpr-derived peptides to ANT-containing proteoliposomes [24]. Importantly, one of the Vpr arginine residues that is required for the interaction with ANT (R77) [S41] is frequently mutated (R77Q) in long-term non-progressors (i.e., HIV-1-carriers who fail to develop signs of CD4^+^ T cell depletion within 15 years after infection) [S116]. This points to the functional relevance of the ANT–Vpr interaction to the development of AIDS. Nonetheless, Vpr has been reported to modulate viral replication independently from its proapoptotic function, presumably via an interaction with host proteins other than ANT [S117]. The minimal cytotoxic domain of Vpr that binds to ANT (aa 67–82) is structured as an amphipathic α-helix [24]. This short cytotoxic domain (Vpr_67–82_) has been recently conjugated with a tumor blood vessel RGD-like “homing” motif. This hybrid peptide is highly efficient in killing human endothelial cells, presumably because it binds to the cell surface, internalizes, and finally interacts with mitochondrial membranes to trigger MMP [S118]. In contrast with Vpr_52–96,_ Vpr_67–82_ induces cell death independently from Bax and Bak, and efficiently overcomes Bcl-2-mediated apoptosis resistance [S118]. The differences in the modes of action of Vpr_52–96_ and Vpr_67–82_ remain elusive. Nonetheless, the design of cell type-targeted mitochondriotoxic peptides, such as those derived from Vpr, has opened tantalizing possibilities for the therapeutic induction of apoptosis (reviewed in [9;S119,S120]).

Hepatitis B virus (HBV) is one of the most prominent etiological agents of chronic liver disease and persistent infection is associated with hepatocellular carcinoma [25;S121]. HBV X protein (HBx) is implicated in viral replication and exhibits an oncogenic potential in animal models [25;S121]. Moreover, HBx sensitizes cells to tumor necrosis factor α (TNFα)-induced apoptosis [S122]. Heterologous expression of HBx in cultured human hepatoma cells results in its mitochondrial accumulation, interaction with VDAC3, and Δψ_m_ dissipation, coupled to a perinuclear redistribution of mitochondria [S123,S124]. Mutagenesis studies revealed that HBx localization to mitochondria and Δψ_m_ dissipation do not depend on its transactivation domain [S125,S126], but require a putative TM region (aa 54–70) of the protein, while two additional amphipathic helical domains (aa 75–88 and 109–131) provide only marginal contributions [S127]. Morevoer, HBx binds to the heat shock protein of 60 kDa (HSP60), a mitochondrial matrix chaperon [S126]. Interestingly, ultrastructural and functional impairment of mitochondria as well as VDAC3 overexpression have been associated with chronic liver disease, further supporting an etiological role for HBx in this context [S128].

Poliovirus (PLV) infection causes paralytic poliomyelitis, an acute disease resulting in flaccid paralysis associated with caspase-dependent apoptosis of motor neurons [S129]. Similar to HBx, the PLV viroporin 2B localizes to mitochondria, induces a perinuclear redistribution of these organelles and alters their morphology, suggesting that 2B might exert proapoptotic effects by directly promoting MMP [S130]. As discussed below, this is not the sole mechanism accounting for PLV-induced neurodegeneration (see the section “Indirect MMP Facilitators”).

Similar perinuclear clustering of mitochondria and Δψ_m_ loss, followed by partial Cyt *c* release and signs of apoptosis (e.g., phosphatidylserine exposure on the outer leaflet of the plasma membrane and chromatin condensation), is observed upon the overexpression of the OrfC protein from the Walleye dermal sarcoma virus (WDSV), a retrovirus causing benign tumors in fish characterized by seasonal regression [S131,S132]. OrfC is a basic protein of 120 aa that localizes to mitochondria, although it does not possess any homology to known mitochondrial targeting sequence (MTS) [S132]. Interestingly, regressing tumors express OrfC at high levels, pointing to the involvement of OrfC-mediated apoptosis in this phenomenon [S131,S132].

Human T lymphotropic virus 1 (HTLV-1) infection is linked with a diverse range of neurodegenerative and lymphoproliferative disorders, notably acute T cell leukemia [26]. The genome of this complex retrovirus codes for typical structural and enzymatic proteins but also for unique regulatory and accessory factors that are involved in both HTLV-1 viral cycle and pathogenesis [S133,S134]. Among these, p13(II) is an 87-aa protein that is targeted to mitochondria, where it promotes a rapid flux of K^+^ and Ca^2+^ across IM together with swelling, Δψ_m_ dissipation, and fragmentation [27;S135]. p13(II) N-terminus includes a short hydrophobic leader peptide, followed by an amphipathic α-helical MTS. Within this region, ten residues are sufficient to target a green fluorescent protein (GFP)–p13(II) fusion to mitochondria [S136], and four arginines form a positively charged patch on one side of the α-helix, which is responsible for its amphipathic properties [S137]. p13(II) expression has been shown to enhance caspase-dependent Fas- and C2 ceramide-induced apoptosis [S138], and to suppress the proliferative and tumorigenic potential of cells transformed by the *myc* and *ras* oncogenes [S135]. The bovine leukemia virus (BLV) is an HTLV-1 homolog causing lymphoproliferative disorders in multiple species [S139]. Similarly to p13(II), BLV accessory protein G4 is localized to both the nucleus and mitochondria, due to an MTS consisting of a hydrophobic region and an amphipathic α-helix [S140]. While G4 is known to alter mitochondrial morphology [S140], its exact role in BLV pathogenesis remains poorly understood. Indeed, whereas G4 exhibits an oncogenic potential both in vitro and in vivo [S141,S142] and G4-deleted BLV variants are characterized by reduced in vivo propagation [S143], BLV-infected peripheral blood mononuclear cells are protected from apoptosis independently from G4 expression [S144]. As a possibility, G4 effects on cellular transformation may rather result from its interaction with farnesyl pyrophosphate synthetase (FPPS) than from the direct modulation of MMP [S145].

PB1-F2 is an 87-aa protein encoded by influenza A virus (IAV) that determines virulence in murine models of infection [28;S146]. While IAVs genetically engineered to lack PB1-F2 replicated normally both in tissue cultures and in mouse lungs, their pathogenicity and associated mortality were greatly diminished as compared to wild-type strains [S146]. PB1-F2 inserts into mitochondrial membranes via a positively charged amphipathic α-helix located in its C-terminus [29]. There, PB1-F2 acquires pore-forming properties similar to those displayed by proapoptotic Bcl-2 family members (e.g., Bax) [S147]. The domain necessary and sufficient for mitochondrial targeting has been mapped to residues 46 to 75 of PB1-F2 [S148]. This region comprises a short hydrophobic region including several basic residues followed by an amphipathic α-helix, and closely resembles the MTS found in Vpr, p13(II) and, G4 [29]. As assessed by glutathione-S-transferase pulldown assays followed by mass spectrometry, the sole mitochondrial proteins interacting with PB1-F2 are the isoform 1 of VDAC (VDAC1) and the isoform 3 of ANT (ANT3) [S149]. Accordingly, PTPC blockers such as BA and CsA prevent PB1-F2-induced MMP and apoptosis [S149]. However, PB1-F2 is capable of destabilizing lipid bilayers in the electric field, implying that the protein may promote MMP by acting directly on mitochondrial membranes [S147].

Altogether, human papillomaviruses (HPVs) are responsible for a broad range of infectious diseases, ranging from anogenital warts (e.g., HPV-6 and -11) to progressive dysplastic-neoplastic lesions of the genital mucosa (e.g., HPV-16 and -18) [S150]. The HPV genome codes for a 90-aa protein encompassing part of the E1 and E4 open reading frames (ORFs), called E1ÎE4 [S151]. In human mature keratinocytes (the natural host for HPV infection), E1ÎE4 binds to cytokeratins, thereby inducing the total collapse of the keratin (but not of tubulin and actin) cytoskeleton [30]. Thereafter (or in cells lacking cytokeratins), E1ÎE4 localizes to mitochondria, due to an N-terminal leucine-rich region [S152]. Via a yet unidentified mechanism, E1ÎE4 displaces mitochondria from microtubules, promoting the aggregation of the organelles in a large perinuclear cluster, followed by Δψ_m_ dissipation and apoptosis [S152].

Both the structural protein VP3 and the non structural protein 2C encoded by the avian encephalomyelitis virus (AEV) promote cell death [S153,S154]. In different cell lines, VP3 localizes to mitochondria and sets off the caspase cascade leading to apoptosis [S153]. Comparable effects result from the expression of 2C, which is a conserved protein of picornaviruses with a role in several steps of the viral life cycle [S154]. The proapoptotic function of 2C is associated with an N-terminal domain of 35 aa (from residues 46 to 80), which has a putative α-helical structure and lies in the proximity of a coiled-coiled domain [S154].

As a last example, the non structural protein 4A (NS4A) from hepatitis C virus (HCV) localizes (at least in part) at mitochondria, thereby causing mitochondrial damage (associated with Δψ_m_ dissipation and Cyt *c* release) and impairing the intracellular distribution of these organelles [S155]. Notably, acute HCV infection often progresses to chronic hepatitis, cirrhosis and hepatocellular carcinoma [S156], and hence it is not surprising that the genome of HVC encodes for several other modulators of apoptosis (see also the sections “Indirect MMP Facilitators” and “Other Antiapoptotic Viral Strategies”) [S156,S157].

### Indirect MMP Facilitators

Multiple proteins encoded by the HIV-1 genome initiate mitochondrial apoptosis in an indirect fashion (Table 1), without a physical interaction with mitochondrial membrane proteins. Thus, Nef stimulates lysosomal membrane permeabilization resulting in the release of cathepsin D from the lysosomal lumen into the cytosol. This triggers the activation of Bax (but not that of Bak) and MMP-dependent cell death [S158,S159].

The HIV-1 envelope glycoprotein complex (Env, constituted by gp140 and gp41) is expressed by infected cells and promotes cell-to-cell fusion by interacting with its receptor/co-receptor complex (CD4/CXCR4 or CD4/CCR5) on the surface of uninfected cells. Env-elicited syncytium formation is followed by MMP after a latency of at least 12 hours [31]. Env triggers MMP through a complex signal transduction pathway that involves the sequential activation of cyclin-dependent kinase 1 (Cdk1), mammalian target of rapamycin (mTOR), p38 MAP kinase, phosphorylation of p53, and p53-dependent transactivation of Puma and Bax [32,33;S160].

Tat, a powerful activator of HIV-1 gene expression, triggers apoptosis of infected and uninfected cells, thereby contributing to the HIV-1-induced neurodegeneration [34;S161–S162]. Transfection with Tat results in its accumulation in mitochondria followed by Δψ_m_ loss, ROS overproduction, and caspase activation [S163]. However, recombinant Tat protein added to cultured cells fails to localize at mitochondria and primarily accumulates in the endosomal compartment, presumably due to its uptake via the endocytic pathway [S164]. Tat associates with the tubulin network through a 4-aa subdomain of its conserved core region, thereby altering microtubule dynamics, promoting the proteasomal degradation of the microtubule-associated protein 2 (MAP2), and activating a mitochondrion-dependent apoptotic pathway [35;S162]. Tat cytotoxicity relies (at least partially) on the proapoptotic activity of the BH3-only protein Bim. In healthy cells, Bim is inactivated through its association with the microtubule-associated dynein motor complex, and Tat liberates Bim from this inhibition [35;S86]. Thus, *bim^−/−^* cells exhibit increased resistance against Tat-induced cell death [35]. Nevertheless, Tat may influence mitochondrial apoptosis through other mechanisms, and contradictory reports suggest that Tat might regulate p53 activity as well as the expression levels of Bax and Bcl-2 [S165–S167].

The HIV-1-encoded protease is required for the viral life cycle because it processes large polypeptide precursors into mature viral proteins. Due to its degenerate substrate specificity, the viral protease promotes the proteolytic activation of caspase-8, as assessed both in vitro and in T cells, which leads to Bid cleavage and mitochondrial apoptosis [36]. Reportedly, the HIV-1 protease would also favor apoptosis and viral replication via the cleavage and inactivation of Bcl-2, which would result in the oxidative stress-dependent activation of NF-κB, a transcription factor required for HIV-1 enhancer activation [S168–S169]. Conversely, high Bcl-2 levels protect cells in vitro and in vivo from the viral protease and prevent apoptosis induced by HIV-1 infection of human lymphocytes, while reducing the yields of viral structural proteins, infectivity, as well as the secretion of tumor necrosis factor β (TNF β) [S169].

Additional viral proteases have been implicated in the induction of apoptosis. Thus, the HCV non structural protein 3 (NS3) participates in a protease complex [S170], and has been reported to induce caspase-8-mediated apoptosis independently from its enzymatic activity [S171]. Upon expression either as single proteins or in combination, PLV proteases 2A (2Apro) and 3C (3Cpro) activate caspase-dependent apoptosis [S172–S174]. However, other mechanisms are involved in the induction of apoptosis by PVs, including **(1)** modulation of antiapoptotic proteins of the Bcl-2 family (e.g., Bcl-X_L_) [S175]; **(2)** JNK-mediated activation of Bax [S176]; and **(3)** MMP promoted by viroporins 2B and 3A (see also the section “Direct Inducers of MMP”) [S130].

Following the infection with human adenoviruses (ADVs), cells exhibit an apoptotic response mediated by the expression of the viral E1A protein. This lethal response can be counteracted by the vBcl-2 E1B-19K (see also the section “Viral Bcl-2 Homologs”) [37]–[39]. Notably, E1A was the first viral protein found to promote apoptosis [37]–[39], via both p53-dependent and -independent mechanisms, the latter involving additional viral factors and in particular products of the E4 gene that are expressed upon E1A-mediated transactivation [S177–S182]. Moreover, E1A sensitizes cells to apoptosis induced by multiple stimuli including death receptor agonists (e.g., FasL, TNFα, and TNF-related apoptosis inducing ligand [TRAIL]) [S183–S185] and nitric oxide (NO) [S186]. Recently, a prominent role for BH3-only proteins (and in particular for Bik) has been reported in ADV-induced apoptosis [40;S187]. Bik is upregulated at the transcriptional level after ADV infection, in a p53-dependent fashion [S188]. Moreover, during the viral life cycle, the proapoptotic activity of Bik is enhanced as a result of an activating phosphorylation. Accordingly, siRNA-mediated depletion of Bik has been shown to dramatically diminish ADV-dependent cell death [S187]. E4orf6, a 34-kDa protein encoded by the adenoviral gene E6, can promote apoptosis by inhibiting the protein phosphatase 2A (PP2A) [41]. PP2A inhibition prolongs the signal of DNA damage emanating from phosphorylated histone H2AX (γH2AX), thereby leading to poly(ADP-ribose) polymerase (PARP) hyperactivation, AIF nuclear translocation, and ultimately cell death [41]. Moreover, PP2A is known to maintain mitochondrial Bcl-2 in an hypophosphorylated form, which allows for its antiapoptotic function [S30]. In this context, PP2A inhibition would also favor the phosphorylation-dependent inactivation of Bcl-2.

E6 and E7 contribute to the transforming properties of high-risk HPVs by targeting p53 to ubiquitin-mediated degradation [42;S189] and by inactivating the retinoblastoma (Rb) protein [43;S190], respectively. E6 (alone or together with E7) has been reported to sensitize cells to different apoptotic stimuli [S191–S193], via mechanisms that may depend [S191,S193] or not [S192] on p53. Similar to E6, E7 has been implicated in the sensitization of cells to cell death induced by growth factor withdrawal [S194], chemotherapeutic agents [S191,S195], and ultraviolet (UV) irradiation [S196].

The vesicular stomatitis virus (VSV) belongs to the family of rhabdoviruses, and its infection is associated with the development of neurological disorders characterized by enhanced neuronal apoptosis [S197,S198]. Thus, VSV-infected cells exhibit an early activation of the mitochondrial pathway of apoptosis [S199], which does not depend on de novo viral protein synthesis nor on viral replication [S199,S200]. Multiple pathways are involved in VSV-induced apoptosis, including **(1)** transcriptional modulation of Bcl-2 family members by wild-type viral matrix (M) protein [S199]; **(2)** activation of the extrinsic apoptotic pathway by mutant M proteins [44;S201]; **(3)** induction of an intracellular oxidative stress [S202]; and **(4)** other mechanisms possibly involving the viral phosphoprotein P [S203]. Interestingly, wild-type and mutant M isoforms exhibit relevant oncolytic properties in vitro and in vivo, thus showing a promising potential as novel biotherapeutic weapons for the treatment of neurological cancers [S204,S205].

Several other viruses have been described to induce apoptosis by hijacking the host machinery that regulates cell death rather than via direct mitochondrial effects. Thus, the foot-and-mouth disease virus (FMDV) capsid protein VP1 induces apoptosis of dendritic cells by an integrin receptor-mediated pathway that activates MMP also via a reduction in the endogenous levels of Bcl-2 [S206]. West Nile virus (WNV)-dependent encephalitis has been associated with the activation of caspase-9 and -3, which would contribute to neuronal apoptosis and local inflammation [45;S207–S209]. At least partially, this would result from the proteolytic activity of the complex constituted by WNV non structural proteins 2B and 3 (NS2B/NS3), which is capable of processing caspase-8, thereby setting off the caspase cascade [S210]. The African swine fever virus (ASFV) promotes apoptosis in vivo in both infected and uninfected cells from the mononuclear phagocytic system [S211]. Such proapoptotic effects are triggered during the process of uncoating independently from viral replication [S212], and presumably depend on the stabilization of the transcription factor p53, leading to the transactivation of cell cycle inhibitors (such as p21^Waf^) and proapoptotic factors (such as Bax) [S213]. Finally, severe acute respiratory syndrome coronavirus (SARS-CoV) protein 7A has been shown to be dispensable for viral replication [S214], but to contribute to virus-induced apoptosis by inhibiting Bcl-X_L_ [46].

## Viral Inhibition of Apoptosis

While the induction of host cell apoptosis may favor viral dissemination at late stages of infection, it is vital for viruses to inhibit PCD at early steps of the infectious cycle, thereby avoiding premature cell death and allowing the virus to replicate. Thus, viruses have developed a battery of Bcl-2 homologs by which they mimic the major antiapoptotic system of host cells (for a recent review, see [S215]). In some instances, such vBcl-2s fail to show significant sequence similarity with their mammalian counterparts, yet exhibit striking structural resemblance. Finally, a number of viral factors inhibit apoptosis via other mechanisms, which do not directly involve the Bcl-2 system (Table 2).

### Viral Bcl-2 Homologs

The “founder” of the viral Bcl-2 homolog (vBcl-2) family is the 19-kDa protein encoded by the adenoviral E1B gene (E1B-19K) [39;S216]. During ADV infection, E1B-19K blocks host cell apoptosis, thereby sustaining viral replication [47]. Moreover, E1B-19K inhibits cell death induced by a plethora of stimuli, including E1A-triggered activation of p53 [38;S177], growth factor deprivation [S217], ligation of TNFR, Fas and TRAIL receptor 1 (TRAIL-R1) at the plasma membrane [48;S218,S219], and heterologous Bax expression [S220]. Similar to Bcl-2, a large fraction of E1B-19K is located at mitochondria where it inhibits apoptosis by sequestering proapoptotic members of the Bcl-2 family such as Bax [49;S220,S221], Bak [S221], and Bnip3 [S222]. In addition, it has been reported that E1B-19K is able to sequester mitochondrial p53 [S223], thereby inhibiting its transcription-independent proapoptotic functions [S23,S24]. Notably, several Bcl-2 family proteins such as Bak [50], Bik [S224], Bnip3 [S222], and the putative BH3-only protein Nip3L [S225] have been originally identified thanks to their interaction with E1B-19K. While E1B-19K and Bcl-2 exhibit limited overall sequence similarity [51], they share short homologous domains that can be exchanged between the two proteins without a significant loss in their antiapoptotic functions [S226,S227]. Accordingly, Bcl-2 and E1B-19K functionally complement each other, and in HeLa cells, Bcl-2 overexpression is able to substitute for the absence of E1B-19K, thereby allowing for productive ADV infection and inhibiting TNFR- and Fas-mediated apoptosis [51].

Human cytomegalovirus (CMV) encodes several proteins that subvert host cell functions in order to favor viral propagation [52]. One of the best characterized among these factors is the product of the CMV gene UL37 (pUL37x1), known also as viral mitochondria-localized inhibitor of apoptosis (vMIA). vMIA, which is required for viral replication, has been shown to inhibit apoptosis triggered by different stimuli, including ligation of death receptors and exposure to cytotoxic agents, as well as infection with a mutant ADV strain lacking the antiapoptotic modulator E1B-19K [S228]. Although vMIA does not share any obvious structural similarity with Bcl-2 nor with its viral homologs (vBcl-2s), vMIA exerts its antiapoptotic activity predominantly by inhibiting MMP at the level of mitochondria [52]. Deletion mutagenesis studies revealed that an N-terminal MTS is necessary and sufficient for vMIA mitochondrial localization, but not for its antiapoptotic activity, which requires an additional C-terminal region of the protein [52;S46,S228]. vMIA can physically interact with Bax, recruit it to mitochondria, and neutralize it [53]. Since vMIA effects on apoptosis are lost in Bax-deficient cells, it appears that vMIA exerts its antiapoptotic functions solely by neutralizing Bax [53]. The structure-function relationship of the Bax/vMIA interaction has been addressed by mutational and computational analyses, suggesting a model in which the overall fold of vMIA closely resembles that of Bcl-X_L_ [S229]. In contrast to Bcl-X_L_, however, it seems that vMIA does not bind to the BH domain of Bax but rather engages in electrostatic interactions involving a region of Bax between its BH2 and BH3 domains [S229]. This region is not conserved in Bak, explaining why vMIA (in contrast to Bcl-X_L_) fails to interact with Bak [53;S229]. Besides its ability to neutralize Bax, vMIA exerts multiple functions, not all of which are directly linked to its MMP-modulatory role. In particular, vMIA **(1)** interacts with ANT and enhances its antiporter activity [54]; **(2)** induces the fragmentation of the mitochondrial network, which might hamper the propagation of Ca^2+^-dependent proapoptotic signals [S230]; **(3)** inhibits the ATP synthasome via an interaction with the mitochondrial inorganic phosphate carrier (PiC, an IM protein), thus reducing mitochondrial ATP generation [54]; and **(4)** induces the release of ER Ca^2+^ stores into the cytosol [S231]. This mobilization of ER Ca^2+^ might have several consequences, including activation of the unfolded protein response, modulation of mitochondrial functions, induction of mitochondrial fission, and protection against proapoptotic signals, through an inhibition in the propagation of Ca^2+^ waves [S231].

Poxviruses cause several diseases of humans and domestic animals, including smallpox, cowpox, sheeppox, fowlpox, and goatpox [S232]. The genome of many poxviruses codes for proteins that, despite the lack of sequence similarity, fold like Bcl-2 and exert similar antiapoptotic properties, thereby belonging de facto to the vBcl-2 family. F1L from vaccinia virus (VACV) was first characterized following the observation that viral strains lacking the serpin CrmA (which acts as a direct inhibitor of caspases [55]) maintained the ability to protect cells from apoptosis [56]. The C-terminus of F1L is composed by a 12-aa TM domain flanked by positively charged lysines and followed by an 8-aa hydrophilic tail. As assessed by mutagenesis studies, this domain (which exhibits slight homology to the C-terminus of Bcl-2) is required for both the mitochondrial targeting and the antiapoptotic function of F1L [S233]. F1L directly interacts with the BH3 domain of proapoptotic Bcl-2 family members, including Bak [57;S234] and Bim [S235], thereby inhibiting Δψ_m_ dissipation and Cyt *c* release induced by diverse stimuli (e.g., staurosporine, Fas crosslinking) [56]. The region of F1L involved in this interaction encompasses aa 64–84 and has limited sequence similarity to known BH3-binding domains [S234]. While F1L has been shown to inhibit mitochondrial translocation and activation of Bax, a direct interaction between F1L and Bax has never been detected, suggesting that F1L acts upstream of Bax activation [S235]. Other poxviruses, including variola virus, monkeypoxvirus, and ectromelia virus, encode functional F1L orthologs, all of which display 95% sequence homology in the C-terminus of the protein [56].

Myxoma virus (MXV) is a leporipoxvirus causing myxomatosis (a highly lethal disease) in the European rabbit. Productive infection by MXV requires the expression of M11L [S236], a 166-aa protein with no defined structural motifs except a putative C-terminal TM domain [S237]. Within this region, six positively charged residues flanking a hydrophobic trait followed by a short positively charged tail constitute an MTS, which is responsible for the targeting of M11L to OM during infection [S237]. This kind of MTS resembles that of some members of the Bcl-2 family [S237]. From its mitochondrial localization, M11L suppresses apoptosis induced by treatment with staurosporine [S237] and the PBR ligand protoporphyrin IX [58], overexpression of Bak [S238], and viral infection [59]. These effects derive from the ability of M11L to constitutively interact with PBR [58], Bak [S238], and Bax [59], and hence to inhibit MPT-dependent MMP as well as Bak/Bax-mediated MOMP. Bax-mediated apoptosis is blocked in MXV-infected cells lacking Bak expression, suggesting that M11L interacts with Bax independently from Bak [59]. M11L has no obvious sequence homology with Bcl-2 or Bcl-X_L_, yet adopts a virtually identical three-dimensional fold [60]. This high level of structural homology allows M11L to associate with a peptide derived from the BH3 domain of Bak with an affinity comparable to that of Bcl-2 and Bcl-X_L_ for the same peptide [60]. Thus, M11L represent a structural mimic of Bcl-2 and hence a bona fide vBcl-2 [61].

N1L is the most potent virulence factor of VACV and is required for productive replication in vivo, as assessed in murine models of mucosal and brain infection [S239,S240]. Preliminary studies indicated that N1L associates with several components of the I-κB kinase (IKK) complex, and hence can interfere with innate immunity signalling mediated by NF-κB and Toll-like receptors (TLRs) [S241,S242]. However, cells infected with recombinant viruses with or without the N1L gene exhibited no difference in NF-κB-dependent gene expression [62], suggesting that another function of N1L underlie its important role in vivo. Indeed, during viral infection, N1L binds to endogenous proapoptotic Bcl-2 family members such as Bid, Bak, and Bax as well as to exogenous Bad and Bax (following transfection-enforced overexpression) [62]. Similar to M11L, N1L shares no sequence homology with cellular proteins yet exhibits a three-dimensional structure that closely mimics that of Bcl-2 and Bcl-X_L_ [S243]. Thus, the surface of N1L possesses a constitutively open groove common to other antiapoptotic members of the Bcl-2 family, which mediates the interaction with BH3-containing proteins [62]. The examples provided by M11L and N1L illustrate the importance of the conservation of structure, rather than of primary amino acid sequence, for the maintenance of protein function along evolution.

Recently, two novel Bcl-2-like inhibitors of apoptosis encoded by poxviruses have been discovered: FPV039 from fowlpox virus (FPV) [63] and ORFV125 from parapoxvirus ORF virus (PPVO) [64]. In human and chicken cells, FPV039 localizes at mitochondria and constitutively interacts with Bak, thereby suppressing apoptosis induced by TNFα and Bak overexpression. Moreover, FPV039 is able to substitute for F1L in inhibiting Bak activation and apoptosis triggered by staurosporine and VACV infection, confirming that FPV039 is a functional homolog of F1L [63]. In UV-irradiated HeLa cells, ORFV125 fully inhibits DNA fragmentation, caspase activation, and Cyt *c* release, but not JNK activation, an event occurring upstream of mitochondria. The mitochondrial localization of ORFV125 is determined by a distinctive C-terminal domain, and is required for its antiapoptotic function. As assessed by bioinformatic analyses, ORFV125 shares sequence and structure similarities with antiapoptotic members of the Bcl-2 family (i.e., Bcl-2, Bcl-X_L_, and Bcl-w). Accordingly, ORFV125 inhibited the UV-induced activation of Bax and Bak, strongly corroborating the idea that ORFV125 represents a novel bona fide member of the vBcl-2 family [64].

Epstein-Barr virus (EBV) is a prominent member of γ-herpesviruses, invading primary resting B lymphocytes to establish a latent infection that eventually culminates in cell transformation [S215]. The potent mitogenic effect of EBV is mediated by the coordinated expression of several gene products, including the apoptotic modulators BALF1 and BHRF1 [S215,S244]. These two factors share sequence and structure homology with Bcl-2 and belong de facto to the growing list of vBcl-2s [65;S245]. While BHRF1 clearly resembles Bcl-2 in its antiapoptotic function [66], the role of BALF1 (which is able to interact with Bax and Bak, and has been proposed as a BHRF1 antagonist) is controversial [65;67]. BALF1 is actively expressed in EBV-positive Burkitt lymphoma's cell lines and nasopharyngeal carcinoma biopsies, and renders cells independent from serum [S246]. This points to a prominent antiapoptotic (rather than proapoptotic) function for BALF1 during EBV-driven tumorigenesis [S246]. As true for other DNA viruses, antiapoptotic proteins appear to be essential for the early phases of the herpesvirus life cycle. However, both proteins are neither expressed nor required once latent infection is established [S215], nor they are essential for in vitro viral replication and transformation of B cells [68;S247]. As assessed by immunoelectron microscopy studies, BHRF1 colocalizes with Bcl-2 at the OM [66],[69]. The C-terminal hydrophobic portion of BHRF1 (which is responsible for BHRF1 targeting to intracellular membranes) exhibits high levels of homology with several members of the Bcl-2 family, in particular with Bcl-2 (38%), Bcl-X_L_ (32%), and Bax (34%) [69;S245]. Moreover, the three-dimensional structure of BHRF1 closely resembles that of Bcl-2 in thus far that it contains two central hydrophobic α-helices that are surrounded by several amphipathic α-helices. In contrast to Bcl-2/Bcl-X_L_, however, BHRF1 is not able to sequestrate and inhibit proapoptotic BH3-only proteins, presumably because it lacks an exposed, pre-formed BH3 binding groove [69;S245]. In spite of this, BHRF1 efficiently suppresses MMP and apoptosis induced by a number of different stimuli, including ligation of death receptors [S248,S249], *c-myc* activation [S250], granzyme B [S251], heterologous viral infection [70], DNA damaging agents [S252], γ irradiation, and multiple chemotherapeutic drugs [S253]. As expected for an antiapoptotic factor mimicking Bcl-2, BHRF1 inhibits TRAIL-induced apoptosis by preventing MMP downstream of the proteolytic activation of Bid [S249]. BHRF1 (but not Bcl-2) overexpression has been shown to promote rapid transit though the cell cycle, suggesting yet another mechanism by which this protein might contribute to EBV-induced tumorigenesis [S254].

BHRF1 is highly conserved at both the sequence and functional levels in different EBV isolates [S255], as well as in EBV analogues infecting chimpanzees (herpesvirus pan) and baboons (herpesvirus papio) [S256]. This not only argues against in vitro studies pointing to a minor role for BHRF1 [68;S247], but rather suggests that BHRF1 plays such a significant, evolutionarily conserved function in vivo that changes in the protein are scarcely tolerated [S256]. Thus, herpesvirus papio BHRF1 (hpoBHRF1) is a 17-kDa protein that shares 64% sequence identity and 79% sequence similarity with BHRF1 from EBV. In functional assays, hpoBHRF1 and BHRF1 similarly protected human keratinocytes against cisplatin-induced apoptosis [S257]. Finally, herpesvirus pan–encoded BHRF1 (hpnBHRF1) has been shown to behave similarly to EBV BHRF1 in protecting a human Burkitt lymphoma cell line (Ramos-BL cells) against apoptosis induced by serum withdrawal, etoposide, and UV irradiation [S258].

vBcl-2s have been identified in other members of the herpesvirus family, i.e., human herpesvirus 8 (HHV-8) [71;S259], murine γ-herpesvirus 68 (γHV-68) [S260,S261], and herpesvirus saimiri (HVS) [72;S262]. HHV-8 ORF16 encodes the so-called Kaposi sarcoma–associated Bcl-2 (KSBcl-2), a polypeptide of 175 residues that shares limited (15%–20%) overall sequence identity with other Bcl-2 homologs (including Bcl-2, Bcl-X_L_, Bax, Bak, and BHRF1) [71]. Interestingly, significant amino acid identity is concentrated in the BH1 and BH2 (but not in the BH3) domains [71]. Moreover, although KSBcl-2 exhibits an overall fold almost identical to that of Bcl-2/Bcl-X_L_, key differences exist in the lengths of helices and loops [73]. Presumably, structure and sequence dissimilarity with Bcl-2 account for the fact that KSBcl-2 neither homodimerizes nor heterodimerizes with other Bcl-2 family members, suggesting that it may have evolved to escape any negative regulatory effects mediated by proapoptotic host proteins from the Bcl-2 family [71],[73].

γHV-68 M11 is a cytoplasmic protein that is expressed in the early-late phase of the lytic infection in vitro [S260,S263], as well as during virus persistence in vivo [S264,S265]. γHV-68 strains harboring mutant M11 show normal lytic replication in vitro and in vivo, yet exhibit reduced splenic latency [S266]. Unlike KSBcl-2, M11 presents only the BH1 (but not the BH2) domain, and hence shares very limited homology with Bcl-2 [S260]. In spite of this, M11 has been shown to inhibit Fas- and TNFα-induced apoptosis in human and murine cell lines [S260,S265], as well as to prevent Bax toxicity in yeast [S267]. Nuclear magnetic resonance studies revealed that M11 shares with other Bcl-2 family members a BH3 binding groove that is able to sequestrate peptides derived from the BH3 domains of Bax and Bak [S267]. Mutation of a conserved arginine and the two adjacent residues within the BH3 binding groove resulted in a correctly folded protein that failed to bind Bax BH3 peptide and to inhibit Bax toxicity in yeast. Moreover, viruses harboring the same mutation exhibited impaired persistent replication and reactivation from latency in vivo [S267]. Altogether, these studies point to a major role of M11 in vivo for the establishment of a persistent viral pool and chronic infection, rather than for viral replication and virulence during acute infection [74].

In contrast to KSBcl-2, HVS ORF16 has been shown to interact with Bax and Bak to inhibit virus-induced apoptosis. While ORF16 exhibits highly conserved BH1 and BH2 domains, it lacks the core sequence of the conserved BH3 domain, suggesting that this portion might be dispensable (at least in some proteins) for antiapoptotic functions [72]. Similar to other vBcl-2s (i.e., BHRF1 and KSBcl-2), ORF16 contains a stretch of conserved hydrophobic residues at its C-terminus, ending with basic amino acids, that may direct its (not yet demonstrated) targeting to intracellular membranes [71]. Interestingly, several herpesvirus-encoded vBcl-2s cannot be converted into proapoptotic factors by activated caspases during PCD (as it occurs to their mammalian counterparts), and hence fail to display any latent proapoptotic activity [75].

In addition to KSBcl-2, HHV-8 codes for yet another protein with limited homology to Bcl-2 [76]. Thus, K7 is a 16-kDa glycoprotein structurally related to a splice variant of human survivin (survivinΔEx3), a mammalian antiapoptotic factor belonging to the inhibitor of apoptosis protein (IAP) family [S268,S269]. Both proteins contain an MTS, an N-terminal region of a baculovirus IAP repeat (BIR) domain, and a putative BH2 domain [76]. The MTS of K7 consists of a single TM hydrophobic region flanked by positively charged residues, and resembles that of M11L from MXV [S270]. K7 efficiently represses apoptosis induced by activation of death receptors (e.g., Fas, TNFR), Bax overexpression and thapsigargin-mediated ER stress [76;S271]. Similarly to other IAPs, K7 binds to and hence inhibits caspase-3 via its BIR domain. However, K7 antiapoptotic effects depend on its BH2 domain, which mediates the interaction of K7 with Bcl-2. Thus, it seems that K7 exerts its functions by bridging effector caspases and Bcl-2, thereby enabling the latter to inhibit caspase activity [76]. Interestingly, K7 has also been shown to modulate intracellular Ca^2+^ concentration and protein stability, by interacting with the cellular Ca^2+^-modulating cyclophilin ligand (CAML) and with a regulator of the ubiquitin system, respectively [S270,S271]. Whereas mutational analyses showed that the interaction between CAML and K7 is required for its antiapoptotic activity [S270], the significance of K7-mediated proteasome regulation remains to be established [S271]. Due to its molecular structure, K7 can be considered either as a viral IAP (vIAP) or as a vBcl-2 [76].

To avoid premature apoptosis of the host cell, ASFV encodes multiple antiapoptotic proteins, including the vBcl-2 family member A179L (also known as 5-HL) [77;S272,S273]. A179L codes for a polypeptide of 21 kDa that contains all known domains associated with Bcl-2 structural and functional features, including those mediating protein-binding (i.e., homo- and heterodimerization) and regulating cell death [S272]. Thus, A179L has been shown to suppress apoptosis induced by multiple stimuli, including growth factor deprivation [S272] and exposure to cytotoxic agents [S274].

### Other Antiapoptotic Viral Strategies

Viruses inhibit host cell apoptosis via a plethora of mechanisms other than vBcl-2s. For instance, the of UL36 gene of CMV encodes the viral inhibitor of caspase-8 activation (vICA), which has been shown to inhibit Fas/CD95-mediated apoptosis by binding to the pro-domain of caspase-8 and preventing its autoproteolytic processing [78]. Although this effect regards in particular the pathway of apoptosis emanating from death receptors, it also avoids the activation of the intrinsic pathway occurring along the caspase-8 → Bid axis [78;S275].

Recently, another mechanism of CMV-mediated inhibition of mitochondrial apoptosis has been discovered [79]. During CMV infection, a 2.7-kilobase virally encoded RNA (β2.7) interacts with the mitochondrial respiratory chain complex I (reduced nicotinamide adenine dinucleotide-ubiquinone oxidoreductase) and prevents the mitochondrial release (that normally would be induced in response to apoptotic stimuli) of the complex I subunit GRIM-19. This stabilizes Δψ_m_ and results in continued ATP production, hence improving the viability of infected cells and favoring the successful completion of the viral life cycle [79]. Thus, CMV employs two distinct strategies to influence mitochondrial respiration of infected cells, namely vMIA-mediated inhibition of the ATP synthasome and stabilization of complex I by β2.7 RNA. As a net result, this combined modulation should not lead to an increase in Δψ_m_, but to decreased mitochondrial ATP generation, correlating with the documented glycolytic switch of CMV-infected cells [S276].

EBV-induced transformation of primary B lymphocytes into continuously proliferating lymphoblastoid cell lines involves proteins other than vBcl-2s [S277]. Among these, the Epstein–Barr nuclear antigens (EBNA) 3A and 3C (EBNA3A and EBNA3C) but not EBNA3B may downregulate the BH3-only protein Bim, and hence reduce the propensity of host cells to undergo apoptosis [S277]. Moreover, the EBNA leader protein (EBNA-LP) has been shown to interfere not only with host cell transcription but also with the apoptotic machinery [80;S278]. Interestingly, it has been suggested that EBNA-LP would interact with Bcl-2 though the HS1-associated protein X-1 (HAX-1) [80], a mitochondrial inhibitor of apoptosis that is regulated by Omi/HtrA2 [S279].

Multiple HCV-encoded proteins have antiapoptotic functions. For instance, the non structural protein 2 (NS2) is a 23-kDa hydrophobic TM protein localized to the ER, whose roles in the viral cycle are not clearly identified [S280,S281]. NS2 is a short-lived protein degraded in the proteasome upon casein kinase 2 (CK2)-mediated phosphorylation [S282]. NS2 reportedly blocks Cyt *c* release and apoptosis induced by CIDE-B [81], a DNA fragmentation factor (DFF)-like cell death effector [S283]. NS2 interaction with CIDE-B involves a 4-aa stretch in NS2 and the C-terminal domain of CIDE-B [81], which is responsible for the homo-/heterodimerization with other CIDE proteins at mitochondria [S283]. As assessed by double immunofluorescence staining, NS2 and CIDE-B exhibit partially overlapping signals in the nuclear proximity, suggesting that the NS2-CIDE-B complex may regulate apoptosis at the mitochondrial level [81]. Another HCV non structural protein, NS5A, is localized at the ER of HCV-infected cells and acts as an antiapoptotic modulator [S284]. NS5A may exert this function by promoting ER stress, which leads to the activation of a pro-survival NF-κB response [S284], or by interacting with the 38-kDa FK506-binding protein (FKBP38) at mitochondria [S285]. Notably, this interaction maps to the aa 148–236 of NS5A, which contains a BH domain [S285]. Finally, HCV structural protein E2 has been shown to inhibit TRAIL-induced apoptosis via a not yet identified mechanism [82].

HHV-8 codes for several antiapoptotic factors other than KSBcl-2 and K7, such as the mitochondrial protein K15 [S286] and K13, a viral Fas-associated death domain-like interleukin 1β converting enzyme (FLICE) inhibitory protein (vFLIP) [83]. The K15 gene contains eight alternatively spliced exons and encodes membrane proteins with a variable number of TM domains plus a common C-terminal region with putative binding sites for SHC homology domains 2 and 3 (SH2 and SH3, respectively), as well as for TNFR-associated factors [S287]. K15, a 45-kDa protein consisting of all eight exons and including 12 TM segments, has been implicated in the regulation of host gene expression, due to its ability to activate several signalling kinases (e.g., JNK, mitogen-activated protein kinases [MAPKs], extracellular signal-regulated kinase [ERK]) and transcription factors (e.g., NF-κB, AP1) [84;S287]. Shorter K15 variants (35- and 23-kDa) exhibit a putative C-terminal MTS, and have been shown to localize to both ER and mitochondria [S286]. Similar to EBNA-LP from EBV, these isoforms of K15 exert an antiapoptotic function by interacting with HAX-1 [S286]. Both K15 and K13 are expressed during latency in HHV-8^+^ primary effusion lymphomas [S288], and K13 is required for the long term survival of infected cells [85]. Thus, K13 promotes the activation of NF-κB via multiple, partially distinct molecular pathways [S289–S292], which in turn lead to **(1)** reduced sensitivity to death receptor-mediated [S293] and intrinsic apoptosis [86]; **(2)** expression of cytokines (e.g., IL-6, IL-8) [S294,S295]; and **(3)** transformation [S296]. Interestingly, K13 expression is necessary and sufficient for the acquisition of the typical spindle shape of HHV-8-infected cells and contributes to their pro-inflammatory phenotype [S297,S298]. Thus, HHV-8 encodes a plethora of factors that participate in virus-associated tumorigenesis by modulating host cell intracellular signalling, proliferation, apoptosis, and immune response [S288].

Various proteins encoded by the E3 transcription unit of ADVs exerts antiapoptotic and immune-modulatory functions, thereby protecting infected cells from cytotoxic T cells and lethal cytokines [S299]. Among these, E3-10.4K and E3-14.5K represent the α and β subunits, respectively, of the receptor internalization and degradation complex (RID), formerly known as E3-10.4/14.5K [S219]. RID inhibits the extrinsic pathway of apoptosis by promoting the internalization and lysosomal degradation of several death receptors, including TNFR [87;S300,S301], Fas [88;S302], TRAIL-R1 [S219,S303], and TRAIL-R2 [S299,S303]. In addition, RID has been shown to target tyrosine kinase receptors, such as the epidermal growth factor receptor (EGFR) [S304]. Both RID subunits are TM proteins oriented with their C-termini in the cytoplasm [S305,S306]. RIDβ contains a C-terminal tyrosine residue that is required for receptor internalization and inhibition of Fas- and TRAIL-induced apoptosis [S307]. RIDα has been reported to undergo O-glycosylation [S308] and phosphorylation on serine residues [S309]. How RIDα post-translational modifications might affect RID antiapoptotic function remains to be established. Mutagenesis studies revealed that the extracellular domain of RIDα is important for the clearance of EGFR from the cell surface, but not for the internalization of death receptors like Fas [S310]. Interestingly, E3-6.7K, another protein encoded by the E3 unit, is specifically implicated in RID-mediated clearance of TRAIL-R2 [S299]. This suggests that additional viral (or cellular) factors might cooperate with RID to determine its target specificity.

Baculoviruses infect insect cells, and possess at least two different classes of proteins by which they control the host apoptotic response [S311]. One of these is represented by p35, a potent inhibitor of metazoan caspases acting via a cleavage-dependent mechanism [89;S312]. p35 mechanism-based inhibition of caspases is the most broadly acting antiapoptotic system known [S313]. Thus, p35 inhibits apoptosis induced by multiple signals in cells from evolutionarily distant organisms such as humans [S314], mice [S315], insects [89,90;S316], nematodes [91,92;S317], and flies [S318]. Following stoichiometric interaction with caspases, the cleavage of p35 at aspartate 87 generates two fragments of 10 and 25 kDa, which remain tightly associated with the enzyme and hence prevent further proteolytic activity [S312]. Recently, p35 has also been shown to inhibit oxidative stress-induced apoptosis, by quenching free radicals at very upstream steps in the apoptotic cascade [S319]. Viral inhibitor of apoptosis proteins (vIAPs) represent the second class of baculoviral antiapoptotic factors [S320]. vIAPs contain a carboxyl ring finger and tandem duplications of a Cys/His motif known as BIR (baculovirus IAP repeat), both of which are crucial for their function [93;S321]. vIAPs homologs have been identified in other viral strains (e.g., A224L from ASFV [S322]) and in multiple metazoan organisms as diverse as humans [S323,S324], mice [94]; flies [95;S325,S326], nematodes [S327,S328], and yeasts [S329,S330]. Both viral and non-viral IAPs inhibit (pro-)caspases (e.g., caspase-3, -7, and -9) downstream of mitochondria [96;S324], thereby suppressing apoptosis mediated by both the extrinsic [S331-S333] and the intrinsic pathways [S331,S334,S335]. Moreover, IAPs have been reported to promote cell survival by activating JNK1 [S336,S337] and the transcription factor NF-κB [97;S338]. Members of the IAP family (e.g., survivin, X chromosome-linked inhibitor of apoptosis protein [XIAP]) are deregulated in most human tumors, and their expression correlates with malignancy, unfavorable prognosis, and resistance to chemo- and radiotherapy [S339-S343]. Thus, IAPs are being used both as diagnostic/prognostic markers [98;S344,S345] and as targets for novel therapeutic approaches [S346-S348].

Recently, the viral mitochondrial antiapoptotic protein (vMAP), encoded by the M8 gene of γHV-68, has been shown to suppress intrinsic apoptosis via a completely novel, dual mechanism [99]. Via its N-terminus, vMAP is able to augment the recruitment of Bcl-2 to mitochondria and to enhance its affinity for BH3-only proapoptotic proteins, thereby suppressing Bax activation. Morevoer, vMAP interacts with VDAC1 via two leucine-rich motifs located in the central and C-terminal parts of the protein, thus repressing staurosporine-induced Cyt *c* release and apoptosis. Interestingly, vMAP is necessary for efficient γHV-68 lytic replication in normal cells (with an intact apoptotic apparatus), but not in *bax^−/−^/ba^−/−^* cells, pointing to a crucial role for apoptosis inhibition during the early steps of the viral life cycle [99].

## Perspectives and Open Questions

We have reviewed the cellular impact of viral infection on cell fate via modulation of mitochondrial apoptosis. While specific cellular and molecular mechanisms have been elucidated for a number of individual proteins (e.g., Vpr, vMIA), a clear scheme of the integrated effects resulting from the expression of whole virus genomes has only recently begun to emerge from transcriptomics and proteomics analyses (for a review, see [100]). Future studies will have to take into account the variability of the host cell and its microenvironmental context (e.g., local inflammation, oxidative stress) as key factors susceptible to modulating the response to specific pathogens. This will undoubtedly be instrumental for the prediction of the general consequences of viral infections, as well as for a more accurate identification of novel therapeutic targets designed to eradicate infectious diseases.

## Accession Numbers

A complete list of accession numbers (UniProtKB/Swiss-Prot knowledgebase, http://www.expasy.org/sprot/) for the proteins discussed in this manuscript can be found online in Text S1.

## Supporting Information

Text S1 List of the accession numbers (UniProtKB/Swiss-Prot Knowledgebase) of all the proteins described in this article.
(0.03 MB DOC)

Text S2 Supplementary references.
(0.12 MB DOC)
