# Supplementary material for: Viral Control of Mitochondrial Apoptosis
Source: PLoS Pathog. 2008 May 30;4(5):e1000018. doi: 10.1371/journal.ppat.1000018 (PMC2376094; doi:10.1371/journal.ppat.1000018)
Supplement: Text S1 — List of the accession numbers (UniProtKB/Swiss-Prot Knowledgebase) of all the proteins described in this article. (0.03 MB DOC) [file ppat.1000018.s001.doc]

**ACCESSION NUMBERS** (online at [http://www.plospathogens.org](http://www.plospathogens.org/))

**UniProtKB/Swiss-Prot Knowledgebase** (<http://www.expasy.org/sprot/>)

Unless otherwise stated in parentheses, host organism is *Homo sapiens*.

2Apro (PLV) , P03300; 2B (PLV), P03300; 2C (AEV), Q9YLS4; 3A (PLV), P03300; 3Cpro (PLV), P03300 ; 7A (SARS-CoV), P59635; A179L (ASFV), P42485; A224L (ASFV), P69180; AIF1, O95831; ANT1, P12235; Nip3L, O60238; Bad, Q92934; Bak1, Q16611; BALF1 (EBV), P03229; Bax, Q07812; Bcl-2, P10415; Bcl-w, Q92843; Bcl-X_L_,, Q07817; BHRF1 (EBV), P03182; Bid, P55957; Bik, Q13323; Bim, O43521; Bmf, Q96LC9; Bnip3, Q12983; CAML, P49069; Capsid (WNV), P06935; Casp-3, P42574; Casp-8, Q14790; Cdk1, P06493; CK, P12532; CK2, P68400/P67870; CIDE-B, Q9UHD4; CrmA (VACV), Q1M1C3; Cyt *c*, P99999; E1B-19K (ADV), P03246; E1^E4 (HPV-16), A7Y3Z3; E1A (ADV), P03255; E2 (HCV), P27958; E3-6.7K (ADV), Q98838; E3-10.4K (ADV), Q6H1B2; E3-14.5K (ADV), Q6H1B1; E4 (ADV), P03239; E4orf6 (ADV), Q2Y0F6; E6 (HPV-16), P03126; E7 (HPV-16), P03129; EBNA3A (EBV), P12977; EBNA3C (EBV), P03204; EBNA-LP (EBV), Q91QU6; EGFR, P00533; Env (HIV-1), P03377; F1L (VACV), P68450; Fas/CD95, P25445; FPV039 (FPV), Q9J5G4; G4 (BLV), A4KC10; GRIM-19, Q9P0J0; HAX-1, O00165; HBx (HBV), P69713; hpoBHRF1 (HPO), Q9WGB5; hpnBHRF1 (HPN), Q9IHR2; HSP60, P10809; JNK, P45983; K7 (HHV-8), Q76RI9; K13 (HHV-8), P88961; K15 (HHV-8), Q91GT9; KSBcl-2 (HHV-8), Q76RI8; IKK complex, O15111/O14920/Q9Y6K9; M (VSV), P03519; M11 (γHV-68), P89884; M11L (MXV), Q85295; mTOR, P42345; Nef (HIV-1), P03406; N1L (VACV), P21054; Noxa, Q13794; NS2 (HCV), P26663; NS3 (HCV), P26664; NS2B/NS3 (WNV), P06935; NS4A (HCV), P26664; NS5A (HCV), P26664; ORF16 (HVS), O40636; OrfC (WDSV), Q88936; ORFV125 (PPVO), Q80G30; p13(II), (HTLV-1) Q9PXZ8; p35 (BCV), P08160; p38 MAPK, Q8IW41; p53, P04637; PB1-F2 (IAV), P0C0U1; phosphoprotein P (VSV), P03520; PP2A, P67775; protease (HIV-1), P04585; vMIA (CMV), A8T7A9; Puma, Q9BXH1; SERCA, O14983; Tat (HIV-1), P04610; TNFR-1, P19438; TRAIL-R1, O00220; TRAIL-R2, O14763; vICA (CMV), P16767; vMAP (γHV-68), O41961; VDAC-1, P21796; VP1 (FMDV), P03306; VP3 (AEV), Q9YLS4; Vpr (HIV-1), P05928.
