# Supplementary material for: Viral Control of Mitochondrial Apoptosis
Source: PLoS Pathog. 2008 May 30;4(5):e1000018. doi: 10.1371/journal.ppat.1000018 (PMC2376094; doi:10.1371/journal.ppat.1000018)
Supplement: Text S2 — Supplementary references. (0.12 MB DOC) [file ppat.1000018.s002.doc]

**SUPPLEMENTAL REFERENCES** (online at [http://www.plospathogens.org](http://www.plospathogens.org/))

1. Jones JD, Dangl JL (2006) The plant immune system. Nature 444: 323-329.
2. Pichlmair A, Reis e Sousa C (2007) Innate recognition of viruses. Immunity 27: 370-383.
3. Hedrick SM (2004) The acquired immune system: a vantage from beneath. Immunity 21: 607-615.
4. Galiana-Arnoux D, Dostert C, Schneemann A, Hoffmann JA, Imler JL (2006) Essential function in vivo for Dicer-2 in host defense against RNA viruses in drosophila. Nat Immunol 7: 590-597.
5. Cullen BR (2006) Is RNA interference involved in intrinsic antiviral immunity in mammals? Nat Immunol 7: 563-567.
6. Welsh RM, Selin LK, Szomolanyi-Tsuda E (2004) Immunological memory to viral infections. Annu Rev Immunol 22: 711-743.
7. Galluzzi L, Maiuri MC, Vitale I, Zischka H, Castedo M, et al. (2007) Cell death modalities: classification and pathophysiological implications. Cell Death Differ 14: 1237-1243.
8. Krammer PH (2000) CD95's deadly mission in the immune system. Nature 407: 789-795.
9. Scaffidi C, Fulda S, Srinivasan A, Friesen C, Li F, et al. (1998) Two CD95 (APO-1/Fas) signaling pathways. Embo J 17: 1675-1687.
10. Jacotot E, Costantini P, Laboureau E, Zamzami N, Susin SA, et al. (1999) Mitochondrial membrane permeabilization during the apoptotic process. Ann N Y Acad Sci 887: 18-30.
11. Reed JC, Kroemer G (2000) Mechanisms of mitochondrial membrane permeabilization. Cell Death Differ 7: 1145.
12. Zamzami N, El Hamel C, Maisse C, Brenner C, Munoz-Pinedo C, et al. (2000) Bid acts on the permeability transition pore complex to induce apoptosis. Oncogene 19: 6342-6350.
13. Liu X, Kim CN, Yang J, Jemmerson R, Wang X (1996) Induction of apoptotic program in cell-free extracts: requirement for dATP and cytochrome c. Cell 86: 147-157.
14. Garrido C, Galluzzi L, Brunet M, Puig PE, Didelot C, et al. (2006) Mechanisms of cytochrome c release from mitochondria. Cell Death Differ 13: 1423-1433.
15. Du C, Fang M, Li Y, Li L, Wang X (2000) Smac, a mitochondrial protein that promotes cytochrome c-dependent caspase activation by eliminating IAP inhibition. Cell 102: 33-42.
16. Susin SA, Lorenzo HK, Zamzami N, Marzo I, Snow BE, et al. (1999) Molecular characterization of mitochondrial apoptosis-inducing factor. Nature 397: 441-446.
17. Modjtahedi N, Giordanetto F, Madeo F, Kroemer G (2006) Apoptosis-inducing factor: vital and lethal. Trends Cell Biol 16: 264-272.
18. Li LY, Luo X, Wang X (2001) Endonuclease G is an apoptotic DNase when released from mitochondria. Nature 412: 95-99.
19. Klohn PC, Soriano ME, Irwin W, Penzo D, Scorrano L, et al. (2003) Early resistance to cell death and to onset of the mitochondrial permeability transition during hepatocarcinogenesis with 2-acetylaminofluorene. Proc Natl Acad Sci U S A 100: 10014-10019.
20. Norenberg MD, Rao KV (2007) The mitochondrial permeability transition in neurologic disease. Neurochem Int 50: 983-997.
21. Er E, Oliver L, Cartron PF, Juin P, Manon S, et al. (2006) Mitochondria as the target of the pro-apoptotic protein Bax. Biochim Biophys Acta 1757: 1301-1311.
22. Mikhailov V, Mikhailova M, Degenhardt K, Venkatachalam MA, White E, et al. (2003) Association of Bax and Bak homo-oligomers in mitochondria. Bax requirement for Bak reorganization and cytochrome c release. J Biol Chem 278: 5367-5376.
23. Mihara M, Erster S, Zaika A, Petrenko O, Chittenden T, et al. (2003) p53 has a direct apoptogenic role at the mitochondria. Mol Cell 11: 577-590.
24. Moll UM, Wolff S, Speidel D, Deppert W (2005) Transcription-independent pro-apoptotic functions of p53. Curr Opin Cell Biol 17: 631-636.
25. Pastorino JG, Tafani M, Farber JL (1999) Tumor necrosis factor induces phosphorylation and translocation of BAD through a phosphatidylinositide-3-OH kinase-dependent pathway. J Biol Chem 274: 19411-19416.
26. Cohen I, Castedo M, Kroemer G (2002) Tantalizing Thanatos: unexpected links in death pathways. Trends Cell Biol 12: 293-295.
27. Schroeter H, Boyd CS, Ahmed R, Spencer JP, Duncan RF, et al. (2003) c-Jun N-terminal kinase (JNK)-mediated modulation of brain mitochondria function: new target proteins for JNK signalling in mitochondrion-dependent apoptosis. Biochem J 372: 359-369.
28. Van Laethem A, Van Kelst S, Lippens S, Declercq W, Vandenabeele P, et al. (2004) Activation of p38 MAPK is required for Bax translocation to mitochondria, cytochrome c release and apoptosis induced by UVB irradiation in human keratinocytes. Faseb J 18: 1946-1948.
29. Dagda RK, Zaucha JA, Wadzinski BE, Strack S (2003) A developmentally regulated, neuron-specific splice variant of the variable subunit Bbeta targets protein phosphatase 2A to mitochondria and modulates apoptosis. J Biol Chem 278: 24976-24985.
30. Tamura Y, Simizu S, Osada H (2004) The phosphorylation status and anti-apoptotic activity of Bcl-2 are regulated by ERK and protein phosphatase 2A on the mitochondria. FEBS Lett 569: 249-255.
31. De Maria R, Rippo MR, Schuchman EH, Testi R (1998) Acidic sphingomyelinase (ASM) is necessary for fas-induced GD3 ganglioside accumulation and efficient apoptosis of lymphoid cells. J Exp Med 187: 897-902.
32. Malisan F, Testi R (1999) Lipid signaling in CD95-mediated apoptosis. FEBS Lett 452: 100-103.
33. Malisan F, Testi R (2002) GD3 ganglioside and apoptosis. Biochim Biophys Acta 1585: 179-187.
34. Choi BM, Pae HO, Jang SI, Kim YM, Chung HT (2002) Nitric oxide as a pro-apoptotic as well as anti-apoptotic modulator. J Biochem Mol Biol 35: 116-126.
35. Petrosillo G, Ruggiero FM, Paradies G (2003) Role of reactive oxygen species and cardiolipin in the release of cytochrome c from mitochondria. Faseb J 17: 2202-2208.
36. Buccellato LJ, Tso M, Akinci OI, Chandel NS, Budinger GR (2004) Reactive oxygen species are required for hyperoxia-induced Bax activation and cell death in alveolar epithelial cells. J Biol Chem 279: 6753-6760.
37. Gomez-Lazaro M, Galindo MF, Melero-Fernandez de Mera RM, Fernandez-Gomez FJ, Concannon CG, et al. (2007) Reactive oxygen species and p38 mitogen-activated protein kinase activate Bax to induce mitochondrial cytochrome c release and apoptosis in response to malonate. Mol Pharmacol 71: 736-743.
38. Boehning D, Patterson RL, Snyder SH (2004) Apoptosis and calcium: new roles for cytochrome c and inositol 1,4,5-trisphosphate. Cell Cycle 3: 252-254.
39. Hajnoczky G, Csordas G, Das S, Garcia-Perez C, Saotome M, et al. (2006) Mitochondrial calcium signalling and cell death: approaches for assessing the role of mitochondrial Ca2+ uptake in apoptosis. Cell Calcium 40: 553-560.
40. Deniaud A, Sharaf El Dein O, Maillier E, Poncet D, Kroemer G, et al. (2007) Endoplasmic reticulum stress induces calcium-dependent permeability transition, mitochondrial outer membrane permeabilization and apoptosis. Oncogene 27: 285-299.
41. Jacotot E, Ravagnan L, Loeffler M, Ferri KF, Vieira HL, et al. (2000) The HIV-1 viral protein R induces apoptosis via a direct effect on the mitochondrial permeability transition pore. J Exp Med 191: 33-46.
42. Boya P, Roques B, Kroemer G (2001) New EMBO members' review: viral and bacterial proteins regulating apoptosis at the mitochondrial level. Embo J 20: 4325-4331.
43. Genestier AL, Michallet MC, Prevost G, Bellot G, Chalabreysse L, et al. (2005) Staphylococcus aureus Panton-Valentine leukocidin directly targets mitochondria and induces Bax-independent apoptosis of human neutrophils. J Clin Invest 115: 3117-3127.
44. Jia J, Wang Y, Zhou L, Jin S (2006) Expression of Pseudomonas aeruginosa toxin ExoS effectively induces apoptosis in host cells. Infect Immun 74: 6557-6570.
45. Gomez-Lazaro M, Galindo MF, Concannon CG, Segura MF, Fernandez-Gomez FJ, et al. (2007) 6-Hydroxydopamine Activates the Mitochondrial Apoptosis Pathway through P38 Mapk-Mediated, P53-Independent Activation of Bax and Puma. J Neurochem.
46. Vieira HL, Belzacq AS, Haouzi D, Bernassola F, Cohen I, et al. (2001) The adenine nucleotide translocator: a target of nitric oxide, peroxynitrite, and 4-hydroxynonenal. Oncogene 20: 4305-4316.
47. Ueda S, Masutani H, Nakamura H, Tanaka T, Ueno M, et al. (2002) Redox control of cell death. Antioxid Redox Signal 4: 405-414.
48. Orrenius S, Gogvadze V, Zhivotovsky B (2007) Mitochondrial oxidative stress: implications for cell death. Annu Rev Pharmacol Toxicol 47: 143-183.
49. Ott M, Gogvadze V, Orrenius S, Zhivotovsky B (2007) Mitochondria, oxidative stress and cell death. Apoptosis 12: 913-922.
50. Scorrano L, Ashiya M, Buttle K, Weiler S, Oakes SA, et al. (2002) A distinct pathway remodels mitochondrial cristae and mobilizes cytochrome c during apoptosis. Dev Cell 2: 55-67.
51. Sun MG, Williams J, Munoz-Pinedo C, Perkins GA, Brown JM, et al. (2007) Correlated three-dimensional light and electron microscopy reveals transformation of mitochondria during apoptosis. Nat Cell Biol 9: 1057-1065.
52. Csordas G, Renken C, Varnai P, Walter L, Weaver D, et al. (2006) Structural and functional features and significance of the physical linkage between ER and mitochondria. J Cell Biol 174: 915-921.
53. Wolter KG, Hsu YT, Smith CL, Nechushtan A, Xi XG, et al. (1997) Movement of Bax from the cytosol to mitochondria during apoptosis. J Cell Biol 139: 1281-1292.
54. Nechushtan A, Smith CL, Lamensdorf I, Yoon SH, Youle RJ (2001) Bax and Bak coalesce into novel mitochondria-associated clusters during apoptosis. J Cell Biol 153: 1265-1276.
55. Basanez G, Sharpe JC, Galanis J, Brandt TB, Hardwick JM, et al. (2002) Bax-type apoptotic proteins porate pure lipid bilayers through a mechanism sensitive to intrinsic monolayer curvature. J Biol Chem 277: 49360-49365.
56. Lucken-Ardjomande S, Martinou JC (2005) Newcomers in the process of mitochondrial permeabilization. J Cell Sci 118: 473-483.
57. Garofalo T, Giammarioli AM, Misasi R, Tinari A, Manganelli V, et al. (2005) Lipid microdomains contribute to apoptosis-associated modifications of mitochondria in T cells. Cell Death Differ 12: 1378-1389.
58. Goonesinghe A, Mundy ES, Smith M, Khosravi-Far R, Martinou JC, et al. (2005) Pro-apoptotic Bid induces membrane perturbation by inserting selected lysolipids into the bilayer. Biochem J 387: 109-118.
59. Shimizu S, Ide T, Yanagida T, Tsujimoto Y (2000) Electrophysiological study of a novel large pore formed by Bax and the voltage-dependent anion channel that is permeable to cytochrome c. J Biol Chem 275: 12321-12325.
60. Shimizu S, Tsujimoto Y (2000) Proapoptotic BH3-only Bcl-2 family members induce cytochrome c release, but not mitochondrial membrane potential loss, and do not directly modulate voltage-dependent anion channel activity. Proc Natl Acad Sci U S A 97: 577-582.
61. Banerjee J, Ghosh S (2004) Bax increases the pore size of rat brain mitochondrial voltage-dependent anion channel in the presence of tBid. Biochem Biophys Res Commun 323: 310-314.
62. Rostovtseva TK, Antonsson B, Suzuki M, Youle RJ, Colombini M, et al. (2004) Bid, but not Bax, regulates VDAC channels. J Biol Chem 279: 13575-13583.
63. Brenner C, Grimm S (2006) The permeability transition pore complex in cancer cell death. Oncogene 25: 4744-4756.
64. Verma YK, Gangenahalli GU, Singh VK, Gupta P, Chandra R, et al. (2006) Cell death regulation by B-cell lymphoma protein. Apoptosis 11: 459-471.
65. Kim R (2005) Unknotting the roles of Bcl-2 and Bcl-xL in cell death. Biochem Biophys Res Commun 333: 336-343.
66. Reed JC (2006) Proapoptotic multidomain Bcl-2/Bax-family proteins: mechanisms, physiological roles, and therapeutic opportunities. Cell Death Differ 13: 1378-1386.
67. Reed JC, Zha H, Aime-Sempe C, Takayama S, Wang HG (1996) Structure-function analysis of Bcl-2 family proteins. Regulators of programmed cell death. Adv Exp Med Biol 406: 99-112.
68. Chan SL, Yu VC (2004) Proteins of the bcl-2 family in apoptosis signalling: from mechanistic insights to therapeutic opportunities. Clin Exp Pharmacol Physiol 31: 119-128.
69. Lanave C, Santamaria M, Saccone C (2004) Comparative genomics: the evolutionary history of the Bcl-2 family. Gene 333: 71-79.
70. Yin XM, Oltvai ZN, Korsmeyer SJ (1994) BH1 and BH2 domains of Bcl-2 are required for inhibition of apoptosis and heterodimerization with Bax. Nature 369: 321-323.
71. Zha H, Aime-Sempe C, Sato T, Reed JC (1996) Proapoptotic protein Bax heterodimerizes with Bcl-2 and homodimerizes with Bax via a novel domain (BH3) distinct from BH1 and BH2. J Biol Chem 271: 7440-7444.
72. Simonen M, Keller H, Heim J (1997) The BH3 domain of Bax is sufficient for interaction of Bax with itself and with other family members and it is required for induction of apoptosis. Eur J Biochem 249: 85-91.
73. Goping IS, Gross A, Lavoie JN, Nguyen M, Jemmerson R, et al. (1998) Regulated targeting of BAX to mitochondria. J Cell Biol 143: 207-215.
74. Froesch BA, Aime-Sempe C, Leber B, Andrews D, Reed JC (1999) Inhibition of p53 transcriptional activity by Bcl-2 requires its membrane-anchoring domain. J Biol Chem 274: 6469-6475.
75. Kaufmann T, Schlipf S, Sanz J, Neubert K, Stein R, et al. (2003) Characterization of the signal that directs Bcl-x(L), but not Bcl-2, to the mitochondrial outer membrane. J Cell Biol 160: 53-64.
76. Denisov AY, Madiraju MS, Chen G, Khadir A, Beauparlant P, et al. (2003) Solution structure of human BCL-w: modulation of ligand binding by the C-terminal helix. J Biol Chem 278: 21124-21128.
77. Heimlich G, McKinnon AD, Bernardo K, Brdiczka D, Reed JC, et al. (2004) Bax-induced cytochrome c release from mitochondria depends on alpha-helices-5 and -6. Biochem J 378: 247-255.
78. van Delft MF, Huang DC (2006) How the Bcl-2 family of proteins interact to regulate apoptosis. Cell Res 16: 203-213.
79. Chen ZX, Pervaiz S (2007) Bcl-2 induces pro-oxidant state by engaging mitochondrial respiration in tumor cells. Cell Death Differ 14: 1617-1627.
80. Pinton P, Rizzuto R (2006) Bcl-2 and Ca2+ homeostasis in the endoplasmic reticulum. Cell Death Differ 13: 1409-1418.
81. Rong Y, Distelhorst CW (2007) Bcl-2 protein family members: versatile regulators of calcium signaling in cell survival and apoptosis. Annu Rev Physiol: in press.
82. Willis SN, Adams JM (2005) Life in the balance: how BH3-only proteins induce apoptosis. Curr Opin Cell Biol 17: 617-625.
83. Griffiths GJ, Dubrez L, Morgan CP, Jones NA, Whitehouse J, et al. (1999) Cell damage-induced conformational changes of the pro-apoptotic protein Bak in vivo precede the onset of apoptosis. J Cell Biol 144: 903-914.
84. Lalier L, Cartron PF, Juin P, Nedelkina S, Manon S, et al. (2007) Bax activation and mitochondrial insertion during apoptosis. Apoptosis 12: 887-896.
85. Nakano K, Vousden KH (2001) PUMA, a novel proapoptotic gene, is induced by p53. Mol Cell 7: 683-694.
86. Puthalakath H, Huang DC, O'Reilly LA, King SM, Strasser A (1999) The proapoptotic activity of the Bcl-2 family member Bim is regulated by interaction with the dynein motor complex. Mol Cell 3: 287-296.
87. Puthalakath H, Villunger A, O'Reilly LA, Beaumont JG, Coultas L, et al. (2001) Bmf: a proapoptotic BH3-only protein regulated by interaction with the myosin V actin motor complex, activated by anoikis. Science 293: 1829-1832.
88. Lei K, Davis RJ (2003) JNK phosphorylation of Bim-related members of the Bcl2 family induces Bax-dependent apoptosis. Proc Natl Acad Sci U S A 100: 2432-2437.
89. Zha J, Weiler S, Oh KJ, Wei MC, Korsmeyer SJ (2000) Posttranslational N-myristoylation of BID as a molecular switch for targeting mitochondria and apoptosis. Science 290: 1761-1765.
90. Lutter M, Fang M, Luo X, Nishijima M, Xie X, et al. (2000) Cardiolipin provides specificity for targeting of tBid to mitochondria. Nat Cell Biol 2: 754-761.
91. Gonzalvez F, Pariselli F, Dupaigne P, Budihardjo I, Lutter M, et al. (2005) tBid interaction with cardiolipin primarily orchestrates mitochondrial dysfunctions and subsequently activates Bax and Bak. Cell Death Differ 12: 614-626.
92. Kim TH, Zhao Y, Ding WX, Shin JN, He X, et al. (2004) Bid-cardiolipin interaction at mitochondrial contact site contributes to mitochondrial cristae reorganization and cytochrome C release. Mol Biol Cell 15: 3061-3072.
93. Liu J, Durrant D, Yang HS, He Y, Whitby FG, et al. (2005) The interaction between tBid and cardiolipin or monolysocardiolipin. Biochem Biophys Res Commun 330: 865-870.
94. Bassik MC, Scorrano L, Oakes SA, Pozzan T, Korsmeyer SJ (2004) Phosphorylation of BCL-2 regulates ER Ca2+ homeostasis and apoptosis. Embo J 23: 1207-1216.
95. Chen R, Valencia I, Zhong F, McColl KS, Roderick HL, et al. (2004) Bcl-2 functionally interacts with inositol 1,4,5-trisphosphate receptors to regulate calcium release from the ER in response to inositol 1,4,5-trisphosphate. J Cell Biol 166: 193-203.
96. Nutt LK, Chandra J, Pataer A, Fang B, Roth JA, et al. (2002) Bax-mediated Ca2+ mobilization promotes cytochrome c release during apoptosis. J Biol Chem 277: 20301-20308.
97. Nutt LK, Pataer A, Pahler J, Fang B, Roth J, et al. (2002) Bax and Bak promote apoptosis by modulating endoplasmic reticular and mitochondrial Ca2+ stores. J Biol Chem 277: 9219-9225.
98. Zamzami N, Larochette N, Kroemer G (2005) Mitochondrial permeability transition in apoptosis and necrosis. Cell Death Differ 12 Suppl 2: 1478-1480.
99. Zoratti M, Szabo I, De Marchi U (2005) Mitochondrial permeability transitions: how many doors to the house? Biochim Biophys Acta 1706: 40-52.
100. Crompton M, Barksby E, Johnson N, Capano M (2002) Mitochondrial intermembrane junctional complexes and their involvement in cell death. Biochimie 84: 143-152.
101. Halestrap AP, Brenner C (2003) The adenine nucleotide translocase: a central component of the mitochondrial permeability transition pore and key player in cell death. Curr Med Chem 10: 1507-1525.
102. Baines CP, Kaiser RA, Sheiko T, Craigen WJ, Molkentin JD (2007) Voltage-dependent anion channels are dispensable for mitochondrial-dependent cell death. Nat Cell Biol 9: 550-555.
103. Petronilli V, Miotto G, Canton M, Brini M, Colonna R, et al. (1999) Transient and long-lasting openings of the mitochondrial permeability transition pore can be monitored directly in intact cells by changes in mitochondrial calcein fluorescence. Biophys J 76: 725-734.
104. Vander Heiden MG, Chandel NS, Li XX, Schumacker PT, Colombini M, et al. (2000) Outer mitochondrial membrane permeability can regulate coupled respiration and cell survival. Proc Natl Acad Sci U S A 97: 4666-4671.
105. Vander Heiden MG, Chandel NS, Schumacker PT, Thompson CB (1999) Bcl-xL prevents cell death following growth factor withdrawal by facilitating mitochondrial ATP/ADP exchange. Mol Cell 3: 159-167.
106. Tsujimoto Y, Shimizu S (2000) VDAC regulation by the Bcl-2 family of proteins. Cell Death Differ 7: 1174-1181.
107. Shimizu S, Konishi A, Kodama T, Tsujimoto Y (2000) BH4 domain of antiapoptotic Bcl-2 family members closes voltage-dependent anion channel and inhibits apoptotic mitochondrial changes and cell death. Proc Natl Acad Sci U S A 97: 3100-3105.
108. Shimizu S, Shinohara Y, Tsujimoto Y (2000) Bax and Bcl-xL independently regulate apoptotic changes of yeast mitochondria that require VDAC but not adenine nucleotide translocator. Oncogene 19: 4309-4318.
109. Sugiyama T, Shimizu S, Matsuoka Y, Yoneda Y, Tsujimoto Y (2002) Activation of mitochondrial voltage-dependent anion channel by apro-apoptotic BH3-only protein Bim. Oncogene 21: 4944-4956.
110. Brenner C, Cadiou H, Vieira HL, Zamzami N, Marzo I, et al. (2000) Bcl-2 and Bax regulate the channel activity of the mitochondrial adenine nucleotide translocator. Oncogene 19: 329-336.
111. Belzacq AS, Vieira HL, Verrier F, Vandecasteele G, Cohen I, et al. (2003) Bcl-2 and Bax modulate adenine nucleotide translocase activity. Cancer Res 63: 541-546.
112. Verrier F, Deniaud A, Lebras M, Metivier D, Kroemer G, et al. (2004) Dynamic evolution of the adenine nucleotide translocase interactome during chemotherapy-induced apoptosis. Oncogene 23: 8049-8064.
113. Stewart SA, Poon B, Jowett JB, Xie Y, Chen IS (1999) Lentiviral delivery of HIV-1 Vpr protein induces apoptosis in transformed cells. Proc Natl Acad Sci U S A 96: 12039-12043.
114. Sabbah EN, Druillennec S, Morellet N, Bouaziz S, Kroemer G, et al. (2006) Interaction between the HIV-1 protein Vpr and the adenine nucleotide translocator. Chem Biol Drug Des 67: 145-154.
115. Muthumani K, Hwang DS, Desai BM, Zhang D, Dayes N, et al. (2002) HIV-1 Vpr induces apoptosis through caspase 9 in T cells and peripheral blood mononuclear cells. J Biol Chem 277: 37820-37831.
116. Lum JJ, Cohen OJ, Nie Z, Weaver JG, Gomez TS, et al. (2003) Vpr R77Q is associated with long-term nonprogressive HIV infection and impaired induction of apoptosis. J Clin Invest 111: 1547-1554.
117. Deniaud A, Brenner C, Kroemer G (2004) Mitochondrial membrane permeabilization by HIV-1 Vpr. Mitochondrion 4: 223-233.
118. Borgne-Sanchez A, Dupont S, Langonne A, Baux L, Lecoeur H, et al. (2007) Targeted Vpr-derived peptides reach mitochondria to induce apoptosis of alphaVbeta3-expressing endothelial cells. Cell Death Differ 14: 422-435.
119. Deniaud A, Hoebeke J, Briand JP, Muller S, Jacotot E, et al. (2006) Peptido-targeting of the mitochondrial transition pore complex for therapeutic apoptosis induction. Curr Pharm Des 12: 4501-4511.
120. Jacotot E, Deniaud A, Borgne-Sanchez A, Touat Z, Briand JP, et al. (2006) Therapeutic peptides: targeting the mitochondrion to modulate apoptosis. Biochim Biophys Acta 1757: 1312-1323.
121. Wang Y, Lau SH, Sham JS, Wu MC, Wang T, et al. (2004) Characterization of HBV integrants in 14 hepatocellular carcinomas: association of truncated X gene and hepatocellular carcinogenesis. Oncogene 23: 142-148.
122. Su F, Schneider RJ (1997) Hepatitis B virus HBx protein sensitizes cells to apoptotic killing by tumor necrosis factor alpha. Proc Natl Acad Sci U S A 94: 8744-8749.
123. Takada S, Shirakata Y, Kaneniwa N, Koike K (1999) Association of hepatitis B virus X protein with mitochondria causes mitochondrial aggregation at the nuclear periphery, leading to cell death. Oncogene 18: 6965-6973.
124. Henkler F, Hoare J, Waseem N, Goldin RD, McGarvey MJ, et al. (2001) Intracellular localization of the hepatitis B virus HBx protein. J Gen Virol 82: 871-882.
125. Rahmani Z, Huh KW, Lasher R, Siddiqui A (2000) Hepatitis B virus X protein colocalizes to mitochondria with a human voltage-dependent anion channel, HVDAC3, and alters its transmembrane potential. J Virol 74: 2840-2846.
126. Tanaka Y, Kanai F, Kawakami T, Tateishi K, Ijichi H, et al. (2004) Interaction of the hepatitis B virus X protein (HBx) with heat shock protein 60 enhances HBx-mediated apoptosis. Biochem Biophys Res Commun 318: 461-469.
127. Huh KW, Siddiqui A (2002) Characterization of the mitochondrial association of hepatitis B virus X protein, HBx. Mitochondrion 1: 349-359.
128. Krahenbuhl S, Stucki J, Reichen J (1989) Mitochondrial function in carbon tetrachloride-induced cirrhosis in the rat. Qualitative and quantitative defects. Biochem Pharmacol 38: 1583-1588.
129. Blondel B, Colbere-Garapin F, Couderc T, Wirotius A, Guivel-Benhassine F (2005) Poliovirus, pathogenesis of poliomyelitis, and apoptosis. Curr Top Microbiol Immunol 289: 25-56.
130. Madan V, Castello A, Carrasco L (2007) Viroporins from RNA viruses induce caspase-dependent apoptosis. Cell Microbiol.
131. LaPierre LA, Casey JW, Holzschu DL (1998) Walleye retroviruses associated with skin tumors and hyperplasias encode cyclin D homologs. J Virol 72: 8765-8771.
132. Nudson WA, Rovnak J, Buechner M, Quackenbush SL (2003) Walleye dermal sarcoma virus Orf C is targeted to the mitochondria. J Gen Virol 84: 375-381.
133. Bartoe JT, Albrecht B, Collins ND, Robek MD, Ratner L, et al. (2000) Functional role of pX open reading frame II of human T-lymphotropic virus type 1 in maintenance of viral loads in vivo. J Virol 74: 1094-1100.
134. Albrecht B, Lairmore MD (2002) Critical role of human T-lymphotropic virus type 1 accessory proteins in viral replication and pathogenesis. Microbiol Mol Biol Rev 66: 396-406, table of contents.
135. Silic-Benussi M, Cavallari I, Zorzan T, Rossi E, Hiraragi H, et al. (2004) Suppression of tumor growth and cell proliferation by p13II, a mitochondrial protein of human T cell leukemia virus type 1. Proc Natl Acad Sci U S A 101: 6629-6634.
136. Ciminale V, Zotti L, D'Agostino DM, Ferro T, Casareto L, et al. (1999) Mitochondrial targeting of the p13II protein coded by the x-II ORF of human T-cell leukemia/lymphotropic virus type I (HTLV-I). Oncogene 18: 4505-4514.
137. D'Agostino DM, Ranzato L, Arrigoni G, Cavallari I, Belleudi F, et al. (2002) Mitochondrial alterations induced by the p13II protein of human T-cell leukemia virus type 1. Critical role of arginine residues. J Biol Chem 277: 34424-34433.
138. Hiraragi H, Michael B, Nair A, Silic-Benussi M, Ciminale V, et al. (2005) Human T-lymphotropic virus type 1 mitochondrion-localizing protein p13II sensitizes Jurkat T cells to Ras-mediated apoptosis. J Virol 79: 9449-9457.
139. Florins A, Gillet N, Asquith B, Boxus M, Burteau C, et al. (2007) Cell dynamics and immune response to BLV infection: a unifying model. Front Biosci 12: 1520-1531.
140. Lefebvre L, Ciminale V, Vanderplasschen A, D'Agostino D, Burny A, et al. (2002) Subcellular localization of the bovine leukemia virus R3 and G4 accessory proteins. J Virol 76: 7843-7854.
141. Kerkhofs P, Heremans H, Burny A, Kettmann R, Willems L (1998) In vitro and in vivo oncogenic potential of bovine leukemia virus G4 protein. J Virol 72: 2554-2559.
142. Schwartz-Cornil I, Chevallier N, Belloc C, Le Rhun D, Laine V, et al. (1997) Bovine leukaemia virus-induced lymphocytosis in sheep is associated with reduction of spontaneous B cell apoptosis. J Gen Virol 78 ( Pt 1): 153-162.
143. Willems L, Kerkhofs P, Dequiedt F, Portetelle D, Mammerickx M, et al. (1994) Attenuation of bovine leukemia virus by deletion of R3 and G4 open reading frames. Proc Natl Acad Sci U S A 91: 11532-11536.
144. Dequiedt F, Hanon E, Kerkhofs P, Pastoret PP, Portetelle D, et al. (1997) Both wild-type and strongly attenuated bovine leukemia viruses protect peripheral blood mononuclear cells from apoptosis. J Virol 71: 630-639.
145. Lefebvre L, Vanderplasschen A, Ciminale V, Heremans H, Dangoisse O, et al. (2002) Oncoviral bovine leukemia virus G4 and human T-cell leukemia virus type 1 p13(II) accessory proteins interact with farnesyl pyrophosphate synthetase. J Virol 76: 1400-1414.
146. Zamarin D, Ortigoza MB, Palese P (2006) Influenza A virus PB1-F2 protein contributes to viral pathogenesis in mice. J Virol 80: 7976-7983.
147. Chanturiya AN, Basanez G, Schubert U, Henklein P, Yewdell JW, et al. (2004) PB1-F2, an influenza A virus-encoded proapoptotic mitochondrial protein, creates variably sized pores in planar lipid membranes. J Virol 78: 6304-6312.
148. Yamada H, Chounan R, Higashi Y, Kurihara N, Kido H (2004) Mitochondrial targeting sequence of the influenza A virus PB1-F2 protein and its function in mitochondria. FEBS Lett 578: 331-336.
149. Zamarin D, Garcia-Sastre A, Xiao X, Wang R, Palese P (2005) Influenza virus PB1-F2 protein induces cell death through mitochondrial ANT3 and VDAC1. PLoS Pathog 1: e4.
150. Wiley D, Masongsong E (2006) Human papillomavirus: the burden of infection. Obstet Gynecol Surv 61: S3-14.
151. Nasseri M, Hirochika R, Broker TR, Chow LT (1987) A human papilloma virus type 11 transcript encoding an E1--E4 protein. Virology 159: 433-439.
152. Raj K, Berguerand S, Southern S, Doorbar J, Beard P (2004) E1 empty set E4 protein of human papillomavirus type 16 associates with mitochondria. J Virol 78: 7199-7207.
153. Liu J, Wei T, Kwang J (2002) Avian encephalomyelitis virus induces apoptosis via major structural protein VP3. Virology 300: 39-49.
154. Liu J, Wei T, Kwang J (2004) Avian encephalomyelitis virus nonstructural protein 2C induces apoptosis by activating cytochrome c/caspase-9 pathway. Virology 318: 169-182.
155. Nomura-Takigawa Y, Nagano-Fujii M, Deng L, Kitazawa S, Ishido S, et al. (2006) Non-structural protein 4A of Hepatitis C virus accumulates on mitochondria and renders the cells prone to undergoing mitochondria-mediated apoptosis. J Gen Virol 87: 1935-1945.
156. Giannini C, Brechot C (2003) Hepatitis C virus biology. Cell Death Differ 10 Suppl 1: S27-38.
157. Machida K, Tsukiyama-Kohara K, Seike E, Tone S, Shibasaki F, et al. (2001) Inhibition of cytochrome c release in Fas-mediated signaling pathway in transgenic mice induced to express hepatitis C viral proteins. J Biol Chem 276: 12140-12146.
158. Laforge M, Petit F, Estaquier J, Senik A (2007) Commitment to apoptosis in CD4(+) T lymphocytes productively infected with human immunodeficiency virus type 1 is initiated by lysosomal membrane permeabilization, itself induced by the isolated expression of the viral protein Nef. J Virol 81: 11426-11440.
159. Rasola A, Gramaglia D, Boccaccio C, Comoglio PM (2001) Apoptosis enhancement by the HIV-1 Nef protein. J Immunol 166: 81-88.
160. Castedo M, Roumier T, Blanco J, Ferri KF, Barretina J, et al. (2002) Sequential involvement of Cdk1, mTOR and p53 in apoptosis induced by the HIV-1 envelope. Embo J 21: 4070-4080.
161. Kruman, II, Nath A, Mattson MP (1998) HIV-1 protein Tat induces apoptosis of hippocampal neurons by a mechanism involving caspase activation, calcium overload, and oxidative stress. Exp Neurol 154: 276-288.
162. Aprea S, Del Valle L, Mameli G, Sawaya BE, Khalili K, et al. (2006) Tubulin-mediated binding of human immunodeficiency virus-1 Tat to the cytoskeleton causes proteasomal-dependent degradation of microtubule-associated protein 2 and neuronal damage. J Neurosci 26: 4054-4062.
163. Macho A, Calzado MA, Jimenez-Reina L, Ceballos E, Leon J, et al. (1999) Susceptibility of HIV-1-TAT transfected cells to undergo apoptosis. Biochemical mechanisms. Oncogene 18: 7543-7551.
164. Ross MF, Filipovska A, Smith RA, Gait MJ, Murphy MP (2004) Cell-penetrating peptides do not cross mitochondrial membranes even when conjugated to a lipophilic cation: evidence against direct passage through phospholipid bilayers. Biochem J 383: 457-468.
165. Sastry KJ, Marin MC, Nehete PN, McConnell K, el-Naggar AK, et al. (1996) Expression of human immunodeficiency virus type I tat results in down-regulation of bcl-2 and induction of apoptosis in hematopoietic cells. Oncogene 13: 487-493.
166. Zauli G, Gibellini D, Caputo A, Bassini A, Negrini M, et al. (1995) The human immunodeficiency virus type-1 Tat protein upregulates Bcl-2 gene expression in Jurkat T-cell lines and primary peripheral blood mononuclear cells. Blood 86: 3823-3834.
167. Li CJ, Wang C, Friedman DJ, Pardee AB (1995) Reciprocal modulations between p53 and Tat of human immunodeficiency virus type 1. Proc Natl Acad Sci U S A 92: 5461-5464.
168. Perkins ND, Edwards NL, Duckett CS, Agranoff AB, Schmid RM, et al. (1993) A cooperative interaction between NF-kappa B and Sp1 is required for HIV-1 enhancer activation. Embo J 12: 3551-3558.
169. Strack PR, Frey MW, Rizzo CJ, Cordova B, George HJ, et al. (1996) Apoptosis mediated by HIV protease is preceded by cleavage of Bcl-2. Proc Natl Acad Sci U S A 93: 9571-9576.
170. Welbourn S, Pause A (2007) The hepatitis C virus NS2/3 protease. Curr Issues Mol Biol 9: 63-69.
171. Prikhod'ko EA, Prikhod'ko GG, Siegel RM, Thompson P, Major ME, et al. (2004) The NS3 protein of hepatitis C virus induces caspase-8-mediated apoptosis independent of its protease or helicase activities. Virology 329: 53-67.
172. Calandria C, Irurzun A, Barco A, Carrasco L (2004) Individual expression of poliovirus 2Apro and 3Cpro induces activation of caspase-3 and PARP cleavage in HeLa cells. Virus Res 104: 39-49.
173. Goldstaub D, Gradi A, Bercovitch Z, Grosmann Z, Nophar Y, et al. (2000) Poliovirus 2A protease induces apoptotic cell death. Mol Cell Biol 20: 1271-1277.
174. Barco A, Feduchi E, Carrasco L (2000) Poliovirus protease 3C(pro) kills cells by apoptosis. Virology 266: 352-360.
175. Calandria C, Lopez-Guerrero JA (2002) Poliovirus modulates Bcl-xl expression in the human U937 promonocytic cell line. Arch Virol 147: 2445-2452.
176. Autret A, Martin-Latil S, Mousson L, Wirotius A, Petit F, et al. (2007) Poliovirus induces Bax-dependent cell death mediated by c-Jun NH2-terminal kinase. J Virol 81: 7504-7516.
177. Debbas M, White E (1993) Wild-type p53 mediates apoptosis by E1A, which is inhibited by E1B. Genes Dev 7: 546-554.
178. White E (1995) Regulation of p53-dependent apoptosis by E1A and E1B. Curr Top Microbiol Immunol 199 ( Pt 3): 34-58.
179. Sabbatini P, Lin J, Levine AJ, White E (1995) Essential role for p53-mediated transcription in E1A-induced apoptosis. Genes Dev 9: 2184-2192.
180. Teodoro JG, Shore GC, Branton PE (1995) Adenovirus E1A proteins induce apoptosis by both p53-dependent and p53-independent mechanisms. Oncogene 11: 467-474.
181. Marcellus RC, Teodoro JG, Wu T, Brough DE, Ketner G, et al. (1996) Adenovirus type 5 early region 4 is responsible for E1A-induced p53-independent apoptosis. J Virol 70: 6207-6215.
182. Lavoie JN, Nguyen M, Marcellus RC, Branton PE, Shore GC (1998) E4orf4, a novel adenovirus death factor that induces p53-independent apoptosis by a pathway that is not inhibited by zVAD-fmk. J Cell Biol 140: 637-645.
183. Routes JM, Ryan S, Clase A, Miura T, Kuhl A, et al. (2000) Adenovirus E1A oncogene expression in tumor cells enhances killing by TNF-related apoptosis-inducing ligand (TRAIL). J Immunol 165: 4522-4527.
184. Perez D, White E (2000) TNF-alpha signals apoptosis through a bid-dependent conformational change in Bax that is inhibited by E1B 19K. Mol Cell 6: 53-63.
185. Cook JL, Potter TA, Bellgrau D, Routes BA (1996) E1A oncogene expression in target cells induces cytolytic susceptibility at a post-recognition stage in the interaction with killer lymphocytes. Oncogene 13: 833-842.
186. Miura TA, Morris K, Ryan S, Cook JL, Routes JM (2003) Adenovirus E1A, not human papillomavirus E7, sensitizes tumor cells to lysis by macrophages through nitric oxide- and TNF-alpha-dependent mechanisms despite up-regulation of 70-kDa heat shock protein. J Immunol 170: 4119-4126.
187. Subramanian T, Vijayalingam S, Lomonosova E, Zhao LJ, Chinnadurai G (2007) Evidence for involvement of BH3-only proapoptotic members in adenovirus-induced apoptosis. J Virol 81: 10486-10495.
188. Mathai JP, Germain M, Marcellus RC, Shore GC (2002) Induction and endoplasmic reticulum location of BIK/NBK in response to apoptotic signaling by E1A and p53. Oncogene 21: 2534-2544.
189. Howley PM, Munger K, Romanczuk H, Scheffner M, Huibregtse JM (1991) Cellular targets of the oncoproteins encoded by the cancer associated human papillomaviruses. Princess Takamatsu Symp 22: 239-248.
190. Zwerschke W, Jansen-Durr P (2000) Cell transformation by the E7 oncoprotein of human papillomavirus type 16: interactions with nuclear and cytoplasmic target proteins. Adv Cancer Res 78: 1-29.
191. Liu Y, McKalip A, Herman B (2000) Human papillomavirus type 16 E6 and HPV-16 E6/E7 sensitize human keratinocytes to apoptosis induced by chemotherapeutic agents: roles of p53 and caspase activation. J Cell Biochem 78: 334-349.
192. Vikhanskaya F, Falugi C, Valente P, Russo P (2002) Human papillomavirus type 16 E6-enhanced susceptibility to apoptosis induced by TNF in A2780 human ovarian cancer cell line. Int J Cancer 97: 732-739.
193. Brown J, Higo H, McKalip A, Herman B (1997) Human papillomavirus (HPV) 16 E6 sensitizes cells to atractyloside-induced apoptosis: role of p53, ICE-like proteases and the mitochondrial permeability transition. J Cell Biochem 66: 245-255.
194. Iglesias M, Yen K, Gaiotti D, Hildesheim A, Stoler MH, et al. (1998) Human papillomavirus type 16 E7 protein sensitizes cervical keratinocytes to apoptosis and release of interleukin-1alpha. Oncogene 17: 1195-1205.
195. Stoppler H, Stoppler MC, Johnson E, Simbulan-Rosenthal CM, Smulson ME, et al. (1998) The E7 protein of human papillomavirus type 16 sensitizes primary human keratinocytes to apoptosis. Oncogene 17: 1207-1214.
196. Lee WT, Lee SH, Carriedo SG, Giffard RG, Yoon YJ, et al. (2002) UV-vulnerability of human papilloma virus type-16 E7-expressing astrocytes is associated with mitochondrial membrane depolarization and caspase-3 activation. Mol Cells 14: 288-294.
197. Sur JH, Allende R, Doster AR (2003) Vesicular stomatitis virus infection and neuropathogenesis in the murine model are associated with apoptosis. Vet Pathol 40: 512-520.
198. Licata JM, Harty RN (2003) Rhabdoviruses and apoptosis. Int Rev Immunol 22: 451-476.
199. Gadaleta P, Perfetti X, Mersich S, Coulombie F (2005) Early activation of the mitochondrial apoptotic pathway in Vesicular Stomatitis virus-infected cells. Virus Res 109: 65-69.
200. Gadaleta P, Vacotto M, Coulombie F (2002) Vesicular stomatitis virus induces apoptosis at early stages in the viral cycle and does not depend on virus replication. Virus Res 86: 87-92.
201. Gaddy DF, Lyles DS (2007) Oncolytic vesicular stomatitis virus induces apoptosis via signaling through PKR, Fas, and Daxx. J Virol 81: 2792-2804.
202. Riva DA, de Molina MC, Rocchetta I, Gerhardt E, Coulombie FC, et al. (2006) Oxidative stress in vero cells infected with vesicular stomatitis virus. Intervirology 49: 294-298.
203. Das SC, Pattnaik AK (2004) Phosphorylation of vesicular stomatitis virus phosphoprotein P is indispensable for virus growth. J Virol 78: 6420-6430.
204. Lin X, Chen X, Wei Y, Zhao J, Fan L, et al. (2007) Efficient inhibition of intraperitoneal human ovarian cancer growth and prolonged survival by gene transfer of vesicular stomatitis virus matrix protein in nude mice. Gynecol Oncol 104: 540-546.
205. Zhang H, Wen Y, Mao B, Gong Q, Qian Z, et al. (2007) Plasmid encoding matrix protein of vesicular stomatitis viruses as an antitumor agent inhibiting rat glioma growth in situ. Exp Oncol 29: 85-93.
206. Jin H, Xiao C, Zhao G, Du X, Yu Y, et al. (2007) Induction of immature dendritic cell apoptosis by foot and mouth disease virus is an integrin receptor mediated event before viral infection. J Cell Biochem 102: 980-991.
207. Samuel MA, Morrey JD, Diamond MS (2007) Caspase 3-dependent cell death of neurons contributes to the pathogenesis of West Nile virus encephalitis. J Virol 81: 2614-2623.
208. Yang JS, Ramanathan MP, Muthumani K, Choo AY, Jin SH, et al. (2002) Induction of inflammation by West Nile virus capsid through the caspase-9 apoptotic pathway. Emerg Infect Dis 8: 1379-1384.
209. Kleinschmidt MC, Michaelis M, Ogbomo H, Doerr HW, Cinatl J, Jr. (2007) Inhibition of apoptosis prevents West Nile virus induced cell death. BMC Microbiol 7: 49.
210. Ramanathan MP, Chambers JA, Pankhong P, Chattergoon M, Attatippaholkun W, et al. (2006) Host cell killing by the West Nile Virus NS2B-NS3 proteolytic complex: NS3 alone is sufficient to recruit caspase-8-based apoptotic pathway. Virology 345: 56-72.
211. Ramiro-Ibanez F, Ortega A, Brun A, Escribano JM, Alonso C (1996) Apoptosis: a mechanism of cell killing and lymphoid organ impairment during acute African swine fever virus infection. J Gen Virol 77 ( Pt 9): 2209-2219.
212. Carrascosa AL, Bustos MJ, Nogal ML, Gonzalez de Buitrago G, Revilla Y (2002) Apoptosis induced in an early step of African swine fever virus entry into vero cells does not require virus replication. Virology 294: 372-382.
213. Granja AG, Nogal ML, Hurtado C, Salas J, Salas ML, et al. (2004) Modulation of p53 cellular function and cell death by African swine fever virus. J Virol 78: 7165-7174.
214. Schaecher SR, Touchette E, Schriewer J, Buller RM, Pekosz A (2007) Severe acute respiratory syndrome coronavirus gene 7 products contribute to virus-induced apoptosis. J Virol 81: 11054-11068.
215. Altmann M, Hammerschmidt W (2005) Epstein-Barr virus provides a new paradigm: a requirement for the immediate inhibition of apoptosis. PLoS Biol 3: e404.
216. White E, Denton A, Stillman B (1988) Role of the adenovirus E1B 19,000-dalton tumor antigen in regulating early gene expression. J Virol 62: 3445-3454.
217. Martinou I, Fernandez PA, Missotten M, White E, Allet B, et al. (1995) Viral proteins E1B19K and p35 protect sympathetic neurons from cell death induced by NGF deprivation. J Cell Biol 128: 201-208.
218. Perez D, White E (1998) E1B 19K inhibits Fas-mediated apoptosis through FADD-dependent sequestration of FLICE. J Cell Biol 141: 1255-1266.
219. Tollefson AE, Toth K, Doronin K, Kuppuswamy M, Doronina OA, et al. (2001) Inhibition of TRAIL-induced apoptosis and forced internalization of TRAIL receptor 1 by adenovirus proteins. J Virol 75: 8875-8887.
220. Han J, Modha D, White E (1998) Interaction of E1B 19K with Bax is required to block Bax-induced loss of mitochondrial membrane potential and apoptosis. Oncogene 17: 2993-3005.
221. Cuconati A, Degenhardt K, Sundararajan R, Anschel A, White E (2002) Bak and Bax function to limit adenovirus replication through apoptosis induction. J Virol 76: 4547-4558.
222. Yasuda M, Theodorakis P, Subramanian T, Chinnadurai G (1998) Adenovirus E1B-19K/BCL-2 interacting protein BNIP3 contains a BH3 domain and a mitochondrial targeting sequence. J Biol Chem 273: 12415-12421.
223. Lomonosova E, Subramanian T, Chinnadurai G (2005) Mitochondrial localization of p53 during adenovirus infection and regulation of its activity by E1B-19K. Oncogene 24: 6796-6808.
224. Han J, Sabbatini P, White E (1996) Induction of apoptosis by human Nbk/Bik, a BH3-containing protein that interacts with E1B 19K. Mol Cell Biol 16: 5857-5864.
225. Ohi N, Tokunaga A, Tsunoda H, Nakano K, Haraguchi K, et al. (1999) A novel adenovirus E1B19K-binding protein B5 inhibits apoptosis induced by Nip3 by forming a heterodimer through the C-terminal hydrophobic region. Cell Death Differ 6: 314-325.
226. Subramanian T, Tarodi B, Chinnadurai G (1995) Functional similarity between adenovirus E1B 19-kDa protein and proteins encoded by Bcl-2 proto-oncogene and Epstein-Barr virus BHRF1 gene. Curr Top Microbiol Immunol 199 ( Pt 1): 153-161.
227. Subramanian T, Boyd JM, Chinnadurai G (1995) Functional substitution identifies a cell survival promoting domain common to adenovirus E1B 19 kDa and Bcl-2 proteins. Oncogene 11: 2403-2409.
228. Goldmacher VS (2002) vMIA, a viral inhibitor of apoptosis targeting mitochondria. Biochimie 84: 177-185.
229. Pauleau AL, Larochette N, Giordanetto F, Scholz SR, Poncet D, et al. (2007) Structure-function analysis of the interaction between Bax and the cytomegalovirus-encoded protein vMIA. Oncogene 26: 7067-7080.
230. McCormick AL, Smith VL, Chow D, Mocarski ES (2003) Disruption of mitochondrial networks by the human cytomegalovirus UL37 gene product viral mitochondrion-localized inhibitor of apoptosis. J Virol 77: 631-641.
231. Sharon-Friling R, Goodhouse J, Colberg-Poley AM, Shenk T (2006) Human cytomegalovirus pUL37x1 induces the release of endoplasmic reticulum calcium stores. Proc Natl Acad Sci U S A 103: 19117-19122.
232. Fenner F (2000) Adventures with poxviruses of vertebrates. FEMS Microbiol Rev 24: 123-133.
233. Stewart TL, Wasilenko ST, Barry M (2005) Vaccinia virus F1L protein is a tail-anchored protein that functions at the mitochondria to inhibit apoptosis. J Virol 79: 1084-1098.
234. Postigo A, Cross JR, Downward J, Way M (2006) Interaction of F1L with the BH3 domain of Bak is responsible for inhibiting vaccinia-induced apoptosis. Cell Death Differ 13: 1651-1662.
235. Taylor JM, Quilty D, Banadyga L, Barry M (2006) The vaccinia virus protein F1L interacts with Bim and inhibits activation of the pro-apoptotic protein Bax. J Biol Chem 281: 39728-39739.
236. Graham KA, Opgenorth A, Upton C, McFadden G (1992) Myxoma virus M11L ORF encodes a protein for which cell surface localization is critical in manifestation of viral virulence. Virology 191: 112-124.
237. Everett H, Barry M, Lee SF, Sun X, Graham K, et al. (2000) M11L: a novel mitochondria-localized protein of myxoma virus that blocks apoptosis of infected leukocytes. J Exp Med 191: 1487-1498.
238. Wang G, Barrett JW, Nazarian SH, Everett H, Gao X, et al. (2004) Myxoma virus M11L prevents apoptosis through constitutive interaction with Bak. J Virol 78: 7097-7111.
239. Billings B, Smith SA, Zhang Z, Lahiri DK, Kotwal GJ (2004) Lack of N1L gene expression results in a significant decrease of vaccinia virus replication in mouse brain. Ann N Y Acad Sci 1030: 297-302.
240. Bartlett N, Symons JA, Tscharke DC, Smith GL (2002) The vaccinia virus N1L protein is an intracellular homodimer that promotes virulence. J Gen Virol 83: 1965-1976.
241. Zhang Z, Abrahams MR, Hunt LA, Suttles J, Marshall W, et al. (2005) The vaccinia virus N1L protein influences cytokine secretion in vitro after infection. Ann N Y Acad Sci 1056: 69-86.
242. DiPerna G, Stack J, Bowie AG, Boyd A, Kotwal G, et al. (2004) Poxvirus protein N1L targets the I-kappaB kinase complex, inhibits signaling to NF-kappaB by the tumor necrosis factor superfamily of receptors, and inhibits NF-kappaB and IRF3 signaling by toll-like receptors. J Biol Chem 279: 36570-36578.
243. Aoyagi M, Zhai D, Jin C, Aleshin AE, Stec B, et al. (2007) Vaccinia virus N1L protein resembles a B cell lymphoma-2 (Bcl-2) family protein. Protein Sci 16: 118-124.
244. Clemens MJ (2006) Epstein-Barr virus: inhibition of apoptosis as a mechanism of cell transformation. Int J Biochem Cell Biol 38: 164-169.
245. Huang Q, Petros AM, Virgin HW, Fesik SW, Olejniczak ET (2003) Solution structure of the BHRF1 protein from Epstein-Barr virus, a homolog of human Bcl-2. J Mol Biol 332: 1123-1130.
246. Cabras G, Decaussin G, Zeng Y, Djennaoui D, Melouli H, et al. (2005) Epstein-Barr virus encoded BALF1 gene is transcribed in Burkitt's lymphoma cell lines and in nasopharyngeal carcinoma's biopsies. J Clin Virol 34: 26-34.
247. Marchini A, Tomkinson B, Cohen JI, Kieff E (1991) BHRF1, the Epstein-Barr virus gene with homology to Bc12, is dispensable for B-lymphocyte transformation and virus replication. J Virol 65: 5991-6000.
248. Kawanishi M (1997) Epstein-Barr virus BHRF1 protein protects intestine 407 epithelial cells from apoptosis induced by tumor necrosis factor alpha and anti-Fas antibody. J Virol 71: 3319-3322.
249. Kawanishi M, Tada-Oikawa S, Kawanishi S (2002) Epstein-Barr virus BHRF1 functions downstream of Bid cleavage and upstream of mitochondrial dysfunction to inhibit TRAIL-induced apoptosis in BJAB cells. Biochem Biophys Res Commun 297: 682-687.
250. Fanidi A, Hancock DC, Littlewood TD (1998) Suppression of c-Myc-induced apoptosis by the Epstein-Barr virus gene product BHRF1. J Virol 72: 8392-8395.
251. Davis JE, Sutton VR, Smyth MJ, Trapani JA (2000) Dependence of granzyme B-mediated cell death on a pathway regulated by Bcl-2 or its viral homolog, BHRF1. Cell Death Differ 7: 973-983.
252. Huang H, Zhou JH, Zhou SM, Hu JH, Pan XH, et al. (1997) Epstein-Barr virus BHRF1 prohibits the cells of nasopharyngeal carcinoma from apoptosis. J Laryngol Otol 111: 1147-1150.
253. McCarthy NJ, Hazlewood SA, Huen DS, Rickinson AB, Williams GT (1996) The Epstein-Barr virus gene BHRF1, a homologue of the cellular oncogene Bcl-2, inhibits apoptosis induced by gamma radiation and chemotherapeutic drugs. Adv Exp Med Biol 406: 83-97.
254. Dawson CW, Dawson J, Jones R, Ward K, Young LS (1998) Functional differences between BHRF1, the Epstein-Barr virus-encoded Bcl-2 homologue, and Bcl-2 in human epithelial cells. J Virol 72: 9016-9024.
255. Khanim F, Dawson C, Meseda CA, Dawson J, Mackett M, et al. (1997) BHRF1, a viral homologue of the Bcl-2 oncogene, is conserved at both the sequence and functional level in different Epstein-Barr virus isolates. J Gen Virol 78 ( Pt 11): 2987-2999.
256. Williams T, Sale D, Hazlewood SA (2001) BHRF1 is highly conserved in primate virus analogues of Epstein-Barr virus. Intervirology 44: 55-58.
257. Meseda CA, Arrand JR, Mackett M (2000) Herpesvirus papio encodes a functional homologue of the Epstein-Barr virus apoptosis suppressor, BHRF1. J Gen Virol 81: 1801-1805.
258. Howell M, Williams T, Hazlewood SA (2005) Herpesvirus pan encodes a functional homologue of BHRF1, the Epstein-Barr virus v-Bcl-2. BMC Microbiol 5: 6.
259. Nicholas J, Ruvolo V, Zong J, Ciufo D, Guo HG, et al. (1997) A single 13-kilobase divergent locus in the Kaposi sarcoma-associated herpesvirus (human herpesvirus 8) genome contains nine open reading frames that are homologous to or related to cellular proteins. J Virol 71: 1963-1974.
260. Wang GH, Garvey TL, Cohen JI (1999) The murine gammaherpesvirus-68 M11 protein inhibits Fas- and TNF-induced apoptosis. J Gen Virol 80 ( Pt 10): 2737-2740.
261. Virgin HWt, Latreille P, Wamsley P, Hallsworth K, Weck KE, et al. (1997) Complete sequence and genomic analysis of murine gammaherpesvirus 68. J Virol 71: 5894-5904.
262. Derfuss T, Fickenscher H, Kraft MS, Henning G, Lengenfelder D, et al. (1998) Antiapoptotic activity of the herpesvirus saimiri-encoded Bcl-2 homolog: stabilization of mitochondria and inhibition of caspase-3-like activity. J Virol 72: 5897-5904.
263. Rochford R, Lutzke ML, Alfinito RS, Clavo A, Cardin RD (2001) Kinetics of murine gammaherpesvirus 68 gene expression following infection of murine cells in culture and in mice. J Virol 75: 4955-4963.
264. Virgin HWt, Presti RM, Li XY, Liu C, Speck SH (1999) Three distinct regions of the murine gammaherpesvirus 68 genome are transcriptionally active in latently infected mice. J Virol 73: 2321-2332.
265. Roy DJ, Ebrahimi BC, Dutia BM, Nash AA, Stewart JP (2000) Murine gammaherpesvirus M11 gene product inhibits apoptosis and is expressed during virus persistence. Arch Virol 145: 2411-2420.
266. de Lima BD, May JS, Marques S, Simas JP, Stevenson PG (2005) Murine gammaherpesvirus 68 bcl-2 homologue contributes to latency establishment in vivo. J Gen Virol 86: 31-40.
267. Loh J, Huang Q, Petros AM, Nettesheim D, van Dyk LF, et al. (2005) A surface groove essential for viral Bcl-2 function during chronic infection in vivo. PLoS Pathog 1: e10.
268. Ambrosini G, Adida C, Altieri DC (1997) A novel anti-apoptosis gene, survivin, expressed in cancer and lymphoma. Nat Med 3: 917-921.
269. Mahotka C, Wenzel M, Springer E, Gabbert HE, Gerharz CD (1999) Survivin-deltaEx3 and survivin-2B: two novel splice variants of the apoptosis inhibitor survivin with different antiapoptotic properties. Cancer Res 59: 6097-6102.
270. Feng P, Park J, Lee BS, Lee SH, Bram RJ, et al. (2002) Kaposi's sarcoma-associated herpesvirus mitochondrial K7 protein targets a cellular calcium-modulating cyclophilin ligand to modulate intracellular calcium concentration and inhibit apoptosis. J Virol 76: 11491-11504.
271. Feng P, Scott CW, Cho NH, Nakamura H, Chung YH, et al. (2004) Kaposi's sarcoma-associated herpesvirus K7 protein targets a ubiquitin-like/ubiquitin-associated domain-containing protein to promote protein degradation. Mol Cell Biol 24: 3938-3948.
272. Afonso CL, Neilan JG, Kutish GF, Rock DL (1996) An African swine fever virus Bc1-2 homolog, 5-HL, suppresses apoptotic cell death. J Virol 70: 4858-4863.
273. Neilan JG, Lu Z, Afonso CL, Kutish GF, Sussman MD, et al. (1993) An African swine fever virus gene with similarity to the proto-oncogene bcl-2 and the Epstein-Barr virus gene BHRF1. J Virol 67: 4391-4394.
274. Revilla Y, Cebrian A, Baixeras E, Martinez C, Vinuela E, et al. (1997) Inhibition of apoptosis by the African swine fever virus Bcl-2 homologue: role of the BH1 domain. Virology 228: 400-404.
275. Andoniou CE, Degli-Esposti MA (2006) Insights into the mechanisms of CMV-mediated interference with cellular apoptosis. Immunol Cell Biol 84: 99-106.
276. Munger J, Bajad SU, Coller HA, Shenk T, Rabinowitz JD (2006) Dynamics of the cellular metabolome during human cytomegalovirus infection. PLoS Pathog 2: e132.
277. Anderton E, Yee J, Smith P, Crook T, White RE, et al. (2007) Two Epstein-Barr virus (EBV) oncoproteins cooperate to repress expression of the proapoptotic tumour-suppressor Bim: clues to the pathogenesis of Burkitt's lymphoma. Oncogene.
278. Inman GJ, Farrell PJ (1995) Epstein-Barr virus EBNA-LP and transcription regulation properties of pRB, p107 and p53 in transfection assays. J Gen Virol 76 ( Pt 9): 2141-2149.
279. Cilenti L, Soundarapandian MM, Kyriazis GA, Stratico V, Singh S, et al. (2004) Regulation of HAX-1 anti-apoptotic protein by Omi/HtrA2 protease during cell death. J Biol Chem 279: 50295-50301.
280. Santolini E, Pacini L, Fipaldini C, Migliaccio G, Monica N (1995) The NS2 protein of hepatitis C virus is a transmembrane polypeptide. J Virol 69: 7461-7471.
281. Yamaga AK, Ou JH (2002) Membrane topology of the hepatitis C virus NS2 protein. J Biol Chem 277: 33228-33234.
282. Franck N, Le Seyec J, Guguen-Guillouzo C, Erdtmann L (2005) Hepatitis C virus NS2 protein is phosphorylated by the protein kinase CK2 and targeted for degradation to the proteasome. J Virol 79: 2700-2708.
283. Inohara N, Koseki T, Chen S, Wu X, Nunez G (1998) CIDE, a novel family of cell death activators with homology to the 45 kDa subunit of the DNA fragmentation factor. Embo J 17: 2526-2533.
284. Waris G, Tardif KD, Siddiqui A (2002) Endoplasmic reticulum (ER) stress: hepatitis C virus induces an ER-nucleus signal transduction pathway and activates NF-kappaB and STAT-3. Biochem Pharmacol 64: 1425-1430.
285. Wang J, Tong W, Zhang X, Chen L, Yi Z, et al. (2006) Hepatitis C virus non-structural protein NS5A interacts with FKBP38 and inhibits apoptosis in Huh7 hepatoma cells. FEBS Lett 580: 4392-4400.
286. Sharp TV, Wang HW, Koumi A, Hollyman D, Endo Y, et al. (2002) K15 protein of Kaposi's sarcoma-associated herpesvirus is latently expressed and binds to HAX-1, a protein with antiapoptotic function. J Virol 76: 802-816.
287. Brinkmann MM, Glenn M, Rainbow L, Kieser A, Henke-Gendo C, et al. (2003) Activation of mitogen-activated protein kinase and NF-kappaB pathways by a Kaposi's sarcoma-associated herpesvirus K15 membrane protein. J Virol 77: 9346-9358.
288. Nicholas J (2007) Human herpesvirus 8-encoded proteins with potential roles in virus-associated neoplasia. Front Biosci 12: 265-281.
289. Field N, Low W, Daniels M, Howell S, Daviet L, et al. (2003) KSHV vFLIP binds to IKK-gamma to activate IKK. J Cell Sci 116: 3721-3728.
290. Matta H, Chaudhary PM (2004) Activation of alternative NF-kappa B pathway by human herpes virus 8-encoded Fas-associated death domain-like IL-1 beta-converting enzyme inhibitory protein (vFLIP). Proc Natl Acad Sci U S A 101: 9399-9404.
291. Guasparri I, Wu H, Cesarman E (2006) The KSHV oncoprotein vFLIP contains a TRAF-interacting motif and requires TRAF2 and TRAF3 for signalling. EMBO Rep 7: 114-119.
292. Matta H, Mazzacurati L, Schamus S, Yang T, Sun Q, et al. (2007) Kaposi's sarcoma-associated herpesvirus (KSHV) oncoprotein K13 bypasses TRAFs and directly interacts with the IkappaB kinase complex to selectively activate NF-kappaB without JNK activation. J Biol Chem 282: 24858-24865.
293. Sarid R, Ben-Moshe T, Kazimirsky G, Weisberg S, Appel E, et al. (2001) vFLIP protects PC-12 cells from apoptosis induced by Sindbis virus: implications for the role of TNF-alpha. Cell Death Differ 8: 1224-1231.
294. An J, Sun Y, Sun R, Rettig MB (2003) Kaposi's sarcoma-associated herpesvirus encoded vFLIP induces cellular IL-6 expression: the role of the NF-kappaB and JNK/AP1 pathways. Oncogene 22: 3371-3385.
295. Sun Q, Matta H, Lu G, Chaudhary PM (2006) Induction of IL-8 expression by human herpesvirus 8 encoded vFLIP K13 via NF-kappaB activation. Oncogene 25: 2717-2726.
296. Sun Q, Zachariah S, Chaudhary PM (2003) The human herpes virus 8-encoded viral FLICE-inhibitory protein induces cellular transformation via NF-kappaB activation. J Biol Chem 278: 52437-52445.
297. Grossmann C, Podgrabinska S, Skobe M, Ganem D (2006) Activation of NF-kappaB by the latent vFLIP gene of Kaposi's sarcoma-associated herpesvirus is required for the spindle shape of virus-infected endothelial cells and contributes to their proinflammatory phenotype. J Virol 80: 7179-7185.
298. Matta H, Surabhi RM, Zhao J, Punj V, Sun Q, et al. (2007) Induction of spindle cell morphology in human vascular endothelial cells by human herpesvirus 8-encoded viral FLICE inhibitory protein K13. Oncogene 26: 1656-1660.
299. Lichtenstein DL, Doronin K, Toth K, Kuppuswamy M, Wold WS, et al. (2004) Adenovirus E3-6.7K protein is required in conjunction with the E3-RID protein complex for the internalization and degradation of TRAIL receptor 2. J Virol 78: 12297-12307.
300. Fessler SP, Chin YR, Horwitz MS (2004) Inhibition of tumor necrosis factor (TNF) signal transduction by the adenovirus group C RID complex involves downregulation of surface levels of TNF receptor 1. J Virol 78: 13113-13121.
301. Gooding LR, Ranheim TS, Tollefson AE, Aquino L, Duerksen-Hughes P, et al. (1991) The 10,400- and 14,500-dalton proteins encoded by region E3 of adenovirus function together to protect many but not all mouse cell lines against lysis by tumor necrosis factor. J Virol 65: 4114-4123.
302. Shisler J, Yang C, Walter B, Ware CF, Gooding LR (1997) The adenovirus E3-10.4K/14.5K complex mediates loss of cell surface Fas (CD95) and resistance to Fas-induced apoptosis. J Virol 71: 8299-8306.
303. Benedict CA, Norris PS, Prigozy TI, Bodmer JL, Mahr JA, et al. (2001) Three adenovirus E3 proteins cooperate to evade apoptosis by tumor necrosis factor-related apoptosis-inducing ligand receptor-1 and -2. J Biol Chem 276: 3270-3278.
304. Tollefson AE, Stewart AR, Yei SP, Saha SK, Wold WS (1991) The 10,400- and 14,500-dalton proteins encoded by region E3 of adenovirus form a complex and function together to down-regulate the epidermal growth factor receptor. J Virol 65: 3095-3105.
305. Krajcsi P, Tollefson AE, Anderson CW, Wold WS (1992) The adenovirus E3 14.5-kilodalton protein, which is required for down-regulation of the epidermal growth factor receptor and prevention of tumor necrosis factor cytolysis, is an integral membrane protein oriented with its C terminus in the cytoplasm. J Virol 66: 1665-1673.
306. Stewart AR, Tollefson AE, Krajcsi P, Yei SP, Wold WS (1995) The adenovirus E3 10.4K and 14.5K proteins, which function to prevent cytolysis by tumor necrosis factor and to down-regulate the epidermal growth factor receptor, are localized in the plasma membrane. J Virol 69: 172-181.
307. Lichtenstein DL, Krajcsi P, Esteban DJ, Tollefson AE, Wold WS (2002) Adenovirus RIDbeta subunit contains a tyrosine residue that is critical for RID-mediated receptor internalization and inhibition of Fas- and TRAIL-induced apoptosis. J Virol 76: 11329-11342.
308. Krajcsi P, Tollefson AE, Wold WS (1992) The E3-14.5K integral membrane protein of adenovirus that is required for down-regulation of the EGF receptor and for prevention of TNF cytolysis is O-glycosylated but not N-glycosylated. Virology 188: 570-579.
309. Krajcsi P, Wold WS (1992) The adenovirus E3-14.5K protein which is required for prevention of TNF cytolysis and for down-regulation of the EGF receptor contains phosphoserine. Virology 187: 492-498.
310. Zanardi TA, Yei S, Lichtenstein DL, Tollefson AE, Wold WS (2003) Distinct domains in the adenovirus E3 RIDalpha protein are required for degradation of Fas and the epidermal growth factor receptor. J Virol 77: 11685-11696.
311. Miller LK (1997) Baculovirus interaction with host apoptotic pathways. J Cell Physiol 173: 178-182.
312. dela Cruz WP, Friesen PD, Fisher AJ (2001) Crystal structure of baculovirus P35 reveals a novel conformational change in the reactive site loop after caspase cleavage. J Biol Chem 276: 32933-32939.
313. Clem RJ (2001) Baculoviruses and apoptosis: the good, the bad, and the ugly. Cell Death Differ 8: 137-143.
314. Beidler DR, Tewari M, Friesen PD, Poirier G, Dixit VM (1995) The baculovirus p35 protein inhibits Fas- and tumor necrosis factor-induced apoptosis. J Biol Chem 270: 16526-16528.
315. Hisahara S, Araki T, Sugiyama F, Yagami K, Suzuki M, et al. (2000) Targeted expression of baculovirus p35 caspase inhibitor in oligodendrocytes protects mice against autoimmune-mediated demyelination. Embo J 19: 341-348.
316. Seshagiri S, Miller LK (1997) Baculovirus inhibitors of apoptosis (IAPs) block activation of Sf-caspase-1. Proc Natl Acad Sci U S A 94: 13606-13611.
317. Bertin J, Mendrysa SM, LaCount DJ, Gaur S, Krebs JF, et al. (1996) Apoptotic suppression by baculovirus P35 involves cleavage by and inhibition of a virus-induced CED-3/ICE-like protease. J Virol 70: 6251-6259.
318. Hay BA, Wolff T, Rubin GM (1994) Expression of baculovirus P35 prevents cell death in Drosophila. Development 120: 2121-2129.
319. Sahdev S, Taneja TK, Mohan M, Sah NK, Khar AK, et al. (2003) Baculoviral p35 inhibits oxidant-induced activation of mitochondrial apoptotic pathway. Biochem Biophys Res Commun 307: 483-490.
320. Clem RJ, Hardwick JM, Miller LK (1996) Anti-apoptotic genes of baculoviruses. Cell Death Differ 3: 9-16.
321. Birnbaum MJ, Clem RJ, Miller LK (1994) An apoptosis-inhibiting gene from a nuclear polyhedrosis virus encoding a polypeptide with Cys/His sequence motifs. J Virol 68: 2521-2528.
322. Nogal ML, Gonzalez de Buitrago G, Rodriguez C, Cubelos B, Carrascosa AL, et al. (2001) African swine fever virus IAP homologue inhibits caspase activation and promotes cell survival in mammalian cells. J Virol 75: 2535-2543.
323. LaCasse EC, Baird S, Korneluk RG, MacKenzie AE (1998) The inhibitors of apoptosis (IAPs) and their emerging role in cancer. Oncogene 17: 3247-3259.
324. Roy N, Deveraux QL, Takahashi R, Salvesen GS, Reed JC (1997) The c-IAP-1 and c-IAP-2 proteins are direct inhibitors of specific caspases. Embo J 16: 6914-6925.
325. Kaiser WJ, Vucic D, Miller LK (1998) The Drosophila inhibitor of apoptosis D-IAP1 suppresses cell death induced by the caspase drICE. FEBS Lett 440: 243-248.
326. Vucic D, Kaiser WJ, Harvey AJ, Miller LK (1997) Inhibition of reaper-induced apoptosis by interaction with inhibitor of apoptosis proteins (IAPs). Proc Natl Acad Sci U S A 94: 10183-10188.
327. Speliotes EK, Uren A, Vaux D, Horvitz HR (2000) The survivin-like C. elegans BIR-1 protein acts with the Aurora-like kinase AIR-2 to affect chromosomes and the spindle midzone. Mol Cell 6: 211-223.
328. Fraser AG, James C, Evan GI, Hengartner MO (1999) Caenorhabditis elegans inhibitor of apoptosis protein (IAP) homologue BIR-1 plays a conserved role in cytokinesis. Curr Biol 9: 292-301.
329. Li F, Flanary PL, Altieri DC, Dohlman HG (2000) Cell division regulation by BIR1, a member of the inhibitor of apoptosis family in yeast. J Biol Chem 275: 6707-6711.
330. Uren AG, Beilharz T, O'Connell MJ, Bugg SJ, van Driel R, et al. (1999) Role for yeast inhibitor of apoptosis (IAP)-like proteins in cell division. Proc Natl Acad Sci U S A 96: 10170-10175.
331. Tamm I, Wang Y, Sausville E, Scudiero DA, Vigna N, et al. (1998) IAP-family protein survivin inhibits caspase activity and apoptosis induced by Fas (CD95), Bax, caspases, and anticancer drugs. Cancer Res 58: 5315-5320.
332. Conway EM, Pollefeyt S, Steiner-Mosonyi M, Luo W, Devriese A, et al. (2002) Deficiency of survivin in transgenic mice exacerbates Fas-induced apoptosis via mitochondrial pathways. Gastroenterology 123: 619-631.
333. Ruemmele FM, Beaulieu JF, O'Connell J, Bennett MW, Seidman EG, et al. (2002) The susceptibility to Fas-induced apoptosis in normal enterocytes is regulated on the level of cIAP1 and 2. Biochem Biophys Res Commun 290: 1308-1314.
334. Cheung HH, Lynn Kelly N, Liston P, Korneluk RG (2006) Involvement of caspase-2 and caspase-9 in endoplasmic reticulum stress-induced apoptosis: a role for the IAPs. Exp Cell Res 312: 2347-2357.
335. Datta R, Oki E, Endo K, Biedermann V, Ren J, et al. (2000) XIAP regulates DNA damage-induced apoptosis downstream of caspase-9 cleavage. J Biol Chem 275: 31733-31738.
336. Sanna MG, da Silva Correia J, Luo Y, Chuang B, Paulson LM, et al. (2002) ILPIP, a novel anti-apoptotic protein that enhances XIAP-mediated activation of JNK1 and protection against apoptosis. J Biol Chem 277: 30454-30462.
337. Sanna MG, da Silva Correia J, Ducrey O, Lee J, Nomoto K, et al. (2002) IAP suppression of apoptosis involves distinct mechanisms: the TAK1/JNK1 signaling cascade and caspase inhibition. Mol Cell Biol 22: 1754-1766.
338. Hofer-Warbinek R, Schmid JA, Stehlik C, Binder BR, Lipp J, et al. (2000) Activation of NF-kappa B by XIAP, the X chromosome-linked inhibitor of apoptosis, in endothelial cells involves TAK1. J Biol Chem 275: 22064-22068.
339. Lopes RB, Gangeswaran R, McNeish IA, Wang Y, Lemoine NR (2007) Expression of the IAP protein family is dysregulated in pancreatic cancer cells and is important for resistance to chemotherapy. Int J Cancer 120: 2344-2352.
340. Kleinberg L, Lie AK, Florenes VA, Nesland JM, Davidson B (2007) Expression of inhibitor-of-apoptosis protein family members in malignant mesothelioma. Hum Pathol 38: 986-994.
341. Al-Joudi FS, Iskandar ZA, Imran AK (2007) Survivin expression correlates with unfavourable prognoses in invasive ductal carcinoma of the breast. Med J Malaysia 62: 6-8.
342. Sohn DM, Kim SY, Baek MJ, Lim CW, Lee MH, et al. (2006) Expression of survivin and clinical correlation in patients with breast cancer. Biomed Pharmacother 60: 289-292.
343. Nakagawa Y, Abe S, Kurata M, Hasegawa M, Yamamoto K, et al. (2006) IAP family protein expression correlates with poor outcome of multiple myeloma patients in association with chemotherapy-induced overexpression of multidrug resistance genes. Am J Hematol 81: 824-831.
344. Andersen MH, Svane IM, Becker JC, Straten PT (2007) The universal character of the tumor-associated antigen survivin. Clin Cancer Res 13: 5991-5994.
345. Duffy MJ, O'Donovan N, Brennan DJ, Gallagher WM, Ryan BM (2007) Survivin: a promising tumor biomarker. Cancer Lett 249: 49-60.
346. Pennati M, Folini M, Zaffaroni N (2007) Targeting survivin in cancer therapy: fulfilled promises and open questions. Carcinogenesis 28: 1133-1139.
347. Fukuda S, Pelus LM (2006) Survivin, a cancer target with an emerging role in normal adult tissues. Mol Cancer Ther 5: 1087-1098.
348. Zaffaroni N, Pennati M, Daidone MG (2005) Survivin as a target for new anticancer interventions. J Cell Mol Med 9: 360-372.
